# Cross disease analysis of co-functional microRNA pairs on a reconstructed network of disease-gene-microRNA tripartite

**DOI:** 10.1186/s12859-017-1605-0

**Published:** 2017-03-24

**Authors:** Hui Peng, Chaowang Lan, Yi Zheng, Gyorgy Hutvagner, Dacheng Tao, Jinyan Li

**Affiliations:** 1 Advanced Analytics Institute, University of Technology Sydney, PO Box 123, Broadway, 2007 NSW Australia; 2 Centre for Health Technologies, University of Technology Sydney, PO Box 123, Broadway, 2007 NSW Australia; 3 School of Information Technologies and the Faculty of Engineering and Information Technologies, University of Sydney, J12/318 Cleveland St, Darlington, 2008 NSW Australia

**Keywords:** Cross-disease analysis, Disease-microRNA associations prediction, Co-functional microRNA pair

## Abstract

**Background:**

MicroRNAs always function cooperatively in their regulation of gene expression. Dysfunctions of these co-functional microRNAs can play significant roles in disease development. We are interested in those multi-disease associated co-functional microRNAs that regulate their common dysfunctional target genes cooperatively in the development of multiple diseases. The research is potentially useful for human disease studies at the transcriptional level and for the study of multi-purpose microRNA therapeutics.

**Methods and results:**

We designed a computational method to detect multi-disease associated co-functional microRNA pairs and conducted cross disease analysis on a reconstructed disease-gene-microRNA (DGR) tripartite network. The construction of the DGR tripartite network is by the integration of newly predicted disease-microRNA associations with those relationships of diseases, microRNAs and genes maintained by existing databases. The prediction method uses a set of reliable negative samples of disease-microRNA association and a pre-computed kernel matrix instead of kernel functions. From this reconstructed DGR tripartite network, multi-disease associated co-functional microRNA pairs are detected together with their common dysfunctional target genes and ranked by a novel scoring method. We also conducted proof-of-concept case studies on cancer-related co-functional microRNA pairs as well as on non-cancer disease-related microRNA pairs.

**Conclusions:**

With the prioritization of the co-functional microRNAs that relate to a series of diseases, we found that the co-function phenomenon is not unusual. We also confirmed that the regulation of the microRNAs for the development of cancers is more complex and have more unique properties than those of non-cancer diseases.

**Electronic supplementary material:**

The online version of this article (doi:10.1186/s12859-017-1605-0) contains supplementary material, which is available to authorized users.

## Background

MicroRNAs (miRNAs), a class of small non-coding RNA of ∼22 nucleotides, are significant regulation molecules for diverse cellular processes such as cell development, proliferation and differentiation [1–7]. Pairs of miRNAs can work cooperatively to regulate an individual gene or a cohort of genes that participate in similar processes [8, 9]. This cooperativity (or co-function) is a frequent regulation mechanism of miRNAs for an enhanced target repression which has exhibited distinctive and fine-tuned target gene expression patterns [10]. Investigation on miRNA cooperativity can systematically understand miRNA functions [11] to detect their potential disease links [12].

Using miRNAs as diagnostic and therapeutic targets, miRNA therapeutics is a promising research area that designs sophisticated strategies to restore or inhibit miRNA expression for the treatment of cancer and other diseases. For example, a therapy with the vector-encoded pair miR-15a and miR-16-1 has been proposed for the treatment of chronic lymphocytic leukaemia (CLL) [13]; The microRNA cluster miR-216a/217 was reported to target genes PTEN and SMAD7 to induce the epithelial-mesenchymal transition, which can promote the drug resistance and recurrence of liver cancer [14]. Such co-functional miRNA pairs are more suitable as drug targets instead of using individual ones. Large scale detection of novel co-functional miRNA pairs is an important pre-step to identify proper miRNA pairs as more effective drug targets. Currently, abundant disease-gene association information are stored in Online Mendelian Inheritance In Man (OMIM) [15] and Comparative Toxicogenomics Database(CTD) [16]; disease-miRNA associations are recorded in miR2Disease [17] and HMDD [18]; and miRNA-target regulations are recorded in miRecord [19] and miRTarBase [20]. Linking and integrating these databases, it can be inferred which diseases are correlated with the same genes or with the same miRNAs, and which miRNAs have the same target disease genes. Our hypothesis is that some of the miRNAs can regulate their common targets cooperatively and have roles in the development of a series of diseases.

**Fig. 1:**
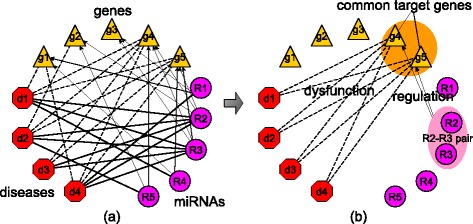
An example: From a DGR tripartite network to a co-functional miRNA pair. The network in panel **a** contains known associations between the genes g1, g2, g3, g4, and g5, the diseases d1, d2, d3, and d4, and the miRNAs R1, R2, R3, and R4. In this example, miRNAs R2 and R3 are both associated with all the four diseases. However, the other three miRNAs are each associated with only one of these diseases. All these four diseases are associated with two common genes g4 and g5. Meanwhile, both of g4 and g5 are the targets of miRNAs R2 and R3. It is believed that R2-R3-g4-g5 in panel **b** may form a functional module that associated with the development of all the four diseases

The focus of this work is on the detection and prioritization of multi-disease associated co-functional miRNA pairs. A multi-disease associated co-functional miRNA pair is a pair of miRNAs whose common target genes are associated with a series of diseases. Here, the definition of co-function for the miRNA pairs is broader than the definition of cooperativity as proposed in [21, 22]. Figure 1 shows an example of multi-disease associated co-functional miRNA pairs detected from a disease-gene-miRNA (DGR) tripartite network. From this example, we can see that multi-disease associated co-functional miRNA pairs may hold a vast mechanism underlying multiple disease development, similarly like the basic cellular functions maintained by housekeeping genes. More importantly, these miRNAs can be considered as the common drug targets of these diseases for the design and development of multi-purpose drugs.

MiRNA co-function mechanisms have attracted intensive research recently [9, 11, 12, 23, 24], with the focus on the analysis of miRNA-target networks or on the analysis of disease-miRNA associations for a specific disease. Our work advances the current research with two steps: (i) We reconstruct a DGR tripartite network through the integration of existing databases with our newly predicted disease-miRNA associations, and (ii) we propose a novel scoring method to prioritize the potential multi-disease associated co-functional miRNA pairs. Since the relationships between the exact miRNAs and diseases are largely unknown, computational methods are required to make prediction of disease-related miRNAs for constructing the disease-miRNA network in the DGR tripartite. For example, network-based or semi-supervised prediction methods [25–27], or the methods via support vector machines [28, 29] can be used among some other prediction methods [30–32]. The key idea in the similarity assessment adopted by most of these methods is that: similar RNAs (functionally similar) are always associated with similar diseases (phenotypically similar, genotypically similar or semantically similar). During the training of the existing prediction methods, the disease-miRNA pairs without known relationships are thought to be ranked at bad positions or are regarded as negative samples directly. As some (probably many) of the unknown disease-miRNA pairs in the training data are true in fact, the false positive rates by the literature methods are high in the prediction of disease related miRNAs. On the other hand, the use of negative samples by the literature methods is straightforward without consideration of gene expression properties of miRNAs.

**Fig. 2:**
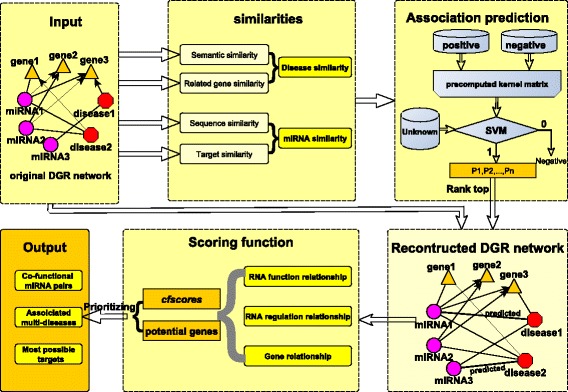
The flowchart of our prediction and scoring method. Our work includes the parts such as material collection, similarity computing, association prediction, network reconstruction, scoring and prioritization of the co-function miRNA pairs and result output

To improve the prediction performance, we propose a new method to make predictions of disease-related miRNAs. Two new ideas are explored. One is the construction of a set of reliable negative samples of disease-miRNA association through miRNA expression comparison between control and diseased subjects. The second idea is the use of precomputed kernel matrix for support vector machines, which can avoid the step to tune the parameters of the kernel functions. The area under the ROC curve(AUC) performance of our method is much superior to the literature methods on bench-marking data sets. Our case studies have demonstrated that our prediction method can also work well even when a disease has no currently known disease-related miRNAs. Combining our predicted disease-miRNA associations with those literature-maintained associations between diseases, miRNAs and genes, we construct a more complete DGR tripartite network to detect and prioritize multi-disease associated co-functional miRNA pairs. Given a miRNA pair, our scoring method *cfscore* considers the function relationship between the two miRNAs, the co-dysexpression of the two miRNAs in the disease tissues and the relationship between the common target genes and the associated diseases of these miRNAs. We are also interested in finding the exact targets dysregulated by the co-functional miRNA pair during the diseases’ development. We call them the co-functional targets of the co-functional miRNA pair. The flowchart of our work is described in Fig. 2.

This method was tested on the cancer and non-cancer disease related DGR tripartite networks. The top 50 multi-disease associated co-functional miRNA pairs were concentrated for deep analysis. We found that most of them were from the same miRNA families or miRNA clusters. The comparison of the co-functional pairs from the two DGR networks suggests that the dysregulation mechanisms of miRNAs in the cancers are more complex. It has also been shown that the analysis of multi-disease associated co-functional miRNAs can help understand the regulation mechanisms of miRNAs in the development of different diseases and thus can provide new knowledge for the diagnosis or treatment of the diseases.

## Results

### Multi-disease associated co-functional miRNA pairs and their common dysfunctional target genes

Two cancer-gene-miRNA tripartite networks were constructed to investigate the performance of our method for detecting and ranking multi-cancer associated co-functional miRNA pairs. As a pre-processing step, we merged the miRCancer database [33] with miR2Disease [17] and HMDD [18], and collected 3655 cancer-miRNA associations between 83 cancers and 503 miRNAs. Connecting these miRNAs and diseases to their associated genes, the first cancer-gene-miRNA tripartite network was constructed. Then, all the 3655 cancer-miRNA associations (as positive samples) and a balanced set of 3655 negative samples of cancer-miRNA association in this tripartite network were used together to train our prediction model for inferring new cancer-miRNA associations. The prediction model was applied to all the un-connected disease-miRNA pairs between the 83 cancers and 503 miRNAs to predict whether some of them have associations or not. When a pair was predicted to have an association between a cancer and a miRNA, a probability was also estimated. A total of 3000 top-ranked associations were added to the first cancer-gene-miRNA tripartite network to form the second cancer-gene-miRNA tripartite network (i.e., a reconstructed network by adding the predicted cancer-miRNA associations). Those associations can be found in the Additional file 1.

On average, the 503 miRNAs are associated with 7 or 13 cancers for the first and the reconstructed network respectively; and there are 2532 and 5634 miRNA pairs in these two networks that have a *cfScore* larger than 0 and that are associated with at least 10 cancers. There are very few literature proving the miRNA pairs can co-function in the development of more than 10 different diseases. To understand whether these miRNA pairs co-function in the development of some of the diseases, we manually searched and examined relevant literature to confirm that the individual miRNAs in the pairs can function cooperatively to regulate the same targets. Of the top-ranked 50 miRNA pairs from our reconstructed network, 40 pairs can be validated to be co-functional pairs by the literature, in comparison with 35 of the top 50 pairs from the first tripartite network. This implies that the addition of the predicted disease-miRNA associations into the tripartite network is useful and effective for the study of co-functional miRNA pairs. Here, we can just confirm these pairs of miRNAs are co-functional miRNA pairs but not multi-disease associated co-functional ones. We could not find any literature that discusses the relationship between miRNAs and a series of diseases.

**Fig. 3:**
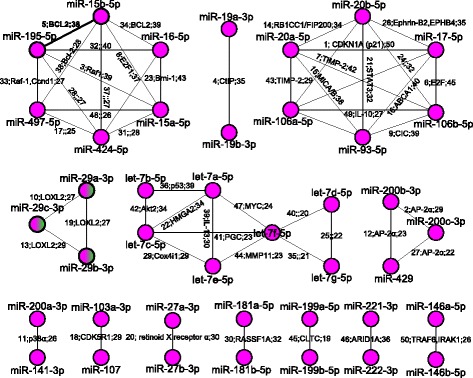
The 50 top-ranked co-functional miRNA pairs from the reconstructed cancer-miRNA-gene network. The labels along the edges illustrate the co-function information of the miRNAs. The first number of each label is the rank of the corresponding pair according to our prioritization method. The following gene symbols are the validated common targets during the co-functioning of the pair of miRNAs. The last number shows the potential diseases that related to this co-function pair

Details of the 50 miRNA pairs are shown in Fig. 3, where on the label of every edge, the first number represents the ranking position of the miRNA pair. If the rank number is followed by one or more gene names, it represents that the miRNA pair is a co-functional pair and has validated common targets. The number at the end of the label is the number of diseases that may associate with this co-functional pair. These multi-cancer associated co-functional miRNA pairs are mostly from the same clusters or families such as from the let-7 family (let-7a ∼7e and miR-98) and the miR-17 ∼92 cluster (miR-17-3p, miR-17-5p, miR-18a, miR-19a, miR-19b, miR-20a and miR-92). It has been known that clustered miRNAs or those miRNAs from the same family are evolved from a common ancestor and can target functionally related genes [34]. Thus, it can be easily understood that miRNAs from the same cluster or family have similar functions and can always function cooperatively. However, not all those miRNAs in the same families or clusters can co-function with each other as their target genes are not completely overlapped. Moreover, some miRNAs that belong to different families or clusters can be co-functional miRNAs. For example, the 17th-ranked pair miR-497-5p-miR-424-5p is a co-functional miRNA pair. However, as recorded by miRBase, miR-424-5p is a member of mir-322 gene family while miR-497-5p stems from the mir-497 family. The pair is also not clustered.

The 5th-ranked pair, miR-15b and miR-195, both belong to the miR-15 family, and both of them can target gene BCL2, an important apoptosis inhibitor. This pair of miRNAs can also work together with another miRNA (miR-16) in regulation [35]. We hypothesize that this co-functional pair may dysregulate their targets cooperatively, leading to the development of 38 different cancers such as prostate cancer (DOID:10283), prostate carcinoma (DOID:10286), stomach cancer (DOID:10534), and breast cancer (DOID:1612). The top three potential common targets of this miRNA pair are genes BCL2 (entrez id:596), CDKN1A (entrez id:1026), and CCND1(entrez id:595). We have verified that these three genes are individually related to most of (68%, 68% or 66%) the 38 cancers. Furthermore, these three genes are all involved in four KEGG [36] pathways: hsa05215: Prostate cancer (*p*-value=1.5E-4), hsa05206: MicroRNAs in cancer (*p*-value=1.7E-3), hsa04151: PI3K-Akt signaling pathway (*p*-value=2.5E-3) and hsa05200: Pathways in cancer (*p*-value=3.2E-3) as revealed by the DAVID functional annotation tool [37, 38]. Moreover, the three genes all have the functions of the cellular response to DNA damage stimulus (GO:0006974, *p*-value=1.4E-4) and response to drug (GO:0042493, *p*-value=4.0E-4), which are important functions for the normal cells. Based on these analysis and evidences, it is suggested that the pair of miR-15b and miR-195 may contribute to the development of all the 38 different types of cancers via a similar regulation mechanism. More details of the discovered miRNA pairs and references are listed in Additional file 2.

We were also interested in the problem of whether the co-functional phenomenon for the non-cancer disease related miRNAs is the same as those of cancers. Thus, we constructed a non-cancer disease related DGR tripartite network containing 1625 non-cancer disease-miRNA associations between 334 miRNAs and 174 diseases extracted from the three existing databases and also containing 1625 predicted associations (Additional file 1). There were just 13 multi-non-cancer-disease associated co-functional miRNA pairs having a *cfscore* bigger than 0 and associating with no less than 10 different diseases. Again, we manually examined these candidate co-functional miRNA pairs. We found that 11 of them can be validated with strong evidence from literature (Additional file 2). Furthermore, 5 of the 13 pairs overlap with the cancer related top 50 miRNA pairs. This indicates that the co-functional mechanism exists not only for the cancer related miRNAs but also for non-cancer disease related miRNAs.

### An in-depth analysis of the 5 overlapping co-functional miRNA pairs

To further understand the regulation mechanism of the co-functional miRNA pairs, we particularly focused on the common targets of the 5 overlapping co-functional pairs (Table 1). The first two columns list the two individual miRNAs in the co-functional miRNA pairs, the third column shows the ranks of those co-functional miRNA pairs. In the forth column, the number of diseases that may relate to the miRNA pairs are displayed, and the last column lists the co-functional targets of these miRNA pairs which are related to multiple diseases. Here, a target gene is ranked higher if it relates to more diseases. It can be seen that even though there are common co-functional miRNA pairs between cancers and non-cancer diseases, the co-functional targets of these miRNA pairs are different from each other. For example, for the two miRNA pairs that both are members of the miR-15 family (miR-15a/b), the top three possible co-functional targets for the non-cancer diseases are IFNG, MTHFR, RARB, while for cancers are BCL2, CDKN1A and CCND1. Meanwhile, there are a lot of genes repeatedly relate to various miRNA pairs such as the last three miRNA pairs from Table 1. Thus these miRNA pairs may function cooperatively and can form a co-functional module. This co-functional module is related to both of multi-cancers and multi-non-cancer diseases.

**Table 1:** The co-functional miRNA pairs and their potential co-functional targets for both cancers and non-cancer diseases

Cancer related co-functional miRNA pairs				
miRNA1	miRNA2	Rank	Cancer numbers	Co-functional targets
miR-15a-5p	miR-15b-5p	8	37	BCL2; CDKN1A; CCND1; VEGFA; MTHFR; IFNG; FGF2; FGFR4; SMAD7; CHEK1
miR-17-5p	miR-20a-5p	1	50	TP53; CCND1; BCL2; CDKN1A; MDM2; VEGFA; MYC; HIF1A; CXCL8; SOD2
miR-29a-3p	miR-29b-3p	19	27	BCL2; MDM2; VEGFA; CASP8; MMP2; PTEN; AKT2; SPARC; VHL; DNMT3B
miR-29a-3p	miR-29c-3p	10	27	BCL2; MDM2; VEGFA; CASP8; MMP2; PTEN; AKT2; SPARC; VHL; DNMT3B
miR-29b-3p	miR-29c-3p	13	29	BCL2; MDM2; VEGFA; CASP8; MMP2; PTEN; VHL; AKT2; SPARC; CCNA2
Non-cancer diseases related co-functional miRNA pairs				
miRNA1	miRNA2	Rank	Disease numbers	Co-functional targets
miR-15a-5p	miR-15b-5p	5	10	IFNG; MTHFR; RARB; BCL2; CSNK1E; JARID2; PDCD1; ALDH3B1; APP; CDC25A
miR-17-5p	miR-20a-5p	2	17	CXCL8; SOD2; BCL2; ESR2; TP53; VEGFA; F3; ITGA2; PTGER4; CCL5
miR-29a-3p	miR-29b-3p	1	20	MMP2; VEGFA; COL3A1; BCL2; FGB; CASP8; FGA; S100B; SPARC; TGFB3
miR-29a-3p	miR-29c-3p	4	13	MMP2; COL3A1; VEGFA; AKT2; CASP8; FGB; MDM2; SGK1; TET2; BCL2
miR-29b-3p	miR-29c-3p	3	14	MMP2; COL3A1; VEGFA; AKT2; CASP8; FGB; MDM2; MMP15; SGK1; MMP24

**Fig. 4:**
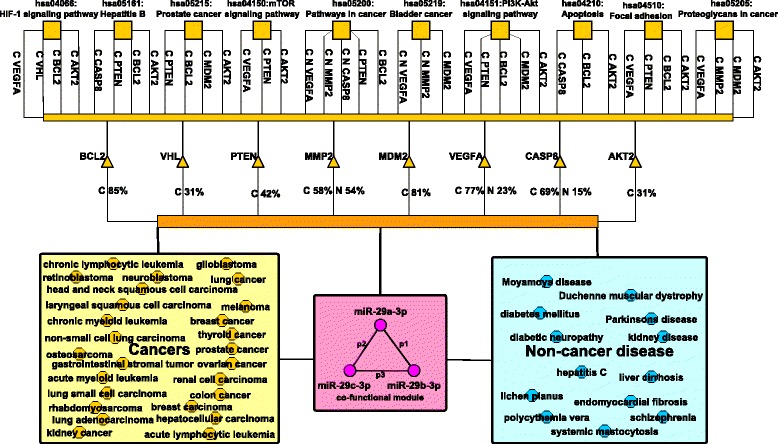
The miR-29a-miR-29b-miR-29c co-function module, their targets and the enrichment analysis of the KEGG pathways. The triangles are the potential common target genes of the miR-29a/b/c co-functional module. Those small squares are the genes enriched pathways. Those disease names in the big squares are the co-functional module related diseases according to our prioritization method

To reveal the detailed regulation mode of these miRNAs associating with multiple cancers and non-cancer diseases, we conducted a deep case analysis. In Fig. 4, the top ten common target genes of each co-functional pair were combined to be a gene set. The DAVID functional annotation tool [37, 38] was applied to analyze these gene sets of the co-functional pairs in the module miR-29a-miR-29b-miR-29c, where the threshold of the pathway enrichment analysis [36] was set as *p*-value ≤0.05 (not the adjusted *p*-value, the following *p*-values are all not adjusted ones). The labels on the edges from the diseases to the genes are the probabilities of genes to be the co-functional targets of the miRNA co-function module. For example, the edge from the diseases to the gene VEGFA has the label of “C 77% N 23%”. This label means that the co-function module may dysregulate the gene VEGFA to contribute to the development of the 26 cancers (C) with the probability of 77%. This gene may also be the common target of the co-functional module during the dysregulation in the development of those 13 non-cancer disease (N) with the probability of 23%. The labels along with the edges connecting the genes and the pathways indicate that the genes from the target gene sets of the diseases (i.e., cancers (C) or non-cancer diseases (N)) associated co-function module can be mapped to the corresponding pathways. For instance, there are three edges connecting the genes with the pathway ‘hsa05219: Bladder cancer’ together with the labels of “C N VEGFA”, “C N MMP2” and “C MDM2”. The labels mean the genes VEGFA, MMP2 and MDM2 from the target gene set of the cancers (C) associated co-function module can be mapped to the Bladder cancer pathway. For the non-cancer diseases (N), only two genes (VEGFA and MMP2) can be mapped to this pathway. Those genes that cannot map to any pathways or those diseases that are not associated with all of the three co-functional pairs are ignored in the figure. The cancer related gene sets can be mapped to many different pathways, we just show the top ten pathways according to their *p*-values.

It is uncovered that the co-functional module mainly dysregulates the ‘hsa05219: Bladder cancer’ and the ‘hsa05200: Pathways in cancer’ to contribute to the development of the 13 non-cancer diseases. The module also regulates eight other pathways (hsa05205, hsa04510, hsa04066, hsa04151, hsa04150, hsa04210, hsa05161 and hsa05215) to involve in the development of the 26 cancers. The cancer developments are more complex with more common genes involved. This observation is consistent with the hypothesis that similar diseases may be related to similar miRNAs and genes. The top three non-cancer disease genes regulated by the co-functional module and mapped to the pathways are MMP2, VEGFA and CASP8, while for the cancers are BCL2, MDM2 and VEGFA. With the gene ontology enrichment analysis, we found that the former three genes have the function of angiogenesis (GO:0001525, *p*-value=1.8E-4), macrophage differentiation (GO:0030225, *p*-value=2.1E-3), negative regulation of cysteine-type endopeptidase activity involved in apoptotic process (GO:0043154, *p*-value=9.0E-3) and response to hypoxia(GO:0001666, *p*-value=2.2E-2). The latter three genes can play roles of cellular response to hypoxia (GO:0071456, *p*-value=5.2E-5), response to iron ion (GO:0010039, *p*-value=2.4E-3), ovarian follicle development (GO:0001541, *p*-value=5.8E-3) and the other related functions. The co-functional module can regulate two same pathways during the development of both the cancers and non-cancer diseases. The possible common targets also have the similar function such as response to hypoxia. These indicate that the miR-29a/b/c regulation module may contribute to the disease development partly via similar dysregulation mechanism. On the other side, the co-functional module may prefer to function by dysregulating the same genes in the development of various cancers rather than those non-cancer diseases. During the carcinogenesis of 26 kinds of cancers, averagely more than 70% of those cancers relate to the dysfunction of the above three genes (BCL2, MDM2 and VEGFA). For the three non-cancer diseases related genes (MMP2, VEGFA and CASP8), the percentage is just around 30%. Those cancers related genes are more likely to involve in the same pathways which indicates the close relationships between their functions. This is mainly due to the fact that cancers are more similar to each other than those non-cancer diseases.

Interestingly, there are a number of literature which have reported the co-function of the miR-29 family members in the development of the cancers such as non-small-cell lung cancer [39], renal cell carcinoma [40], breast cancer [41], ovarian cancer [42] and others types of cancers [43]. Furthermore, the MYC-mediated miR-29 repression mechanism for the therapy of aggressive B-cell malignancies (B-cell malignancies is the synonym of chronic lymphocytic leukemia according to Medical Subject Headings (MeSH) [44]) by applying the HDAC3 and EZH2 as therapeutic targets [45] was reported. Another report in 2015 also discussed the adoption of miR-29s (miR-29a/b/c) as candidate epi-therapeutics for curing hematologic malignancies [46]. According to our findings and literature, we can claim that it is reasonable to consider miR-29a/b/c as potential drug targets for the treatment of multiple cancers.

### The predicted miRNAs that are related to breast and prostate cancer: case studies

In this section, we report details of the predicted miRNAs which are likely related to breast cancer and prostate cancer. Breast cancer is the leading type of cancer in women, accounting for 25% of all women cancer patients [47]. Prostate cancer is the second most common type of cancer and the fifth leading cause of cancer-related death in men [47]. We have taken the following three steps for this case study: (1) the prediction model was trained on the RLSMDA data set of disease-miRNA associations [26] which contains 1184 disease-miRNA associations; (2) the prediction model was applied to make predictions for those disease-miRNA pairs whose relationships were unknown in this data set; (3) the positively predicted disease-miRNA pairs were evaluated using the latest version of databases such as miRCancer [33], miR2Disease [17] and HMDD [18], which stores newer disease-miRNA associations than the RLSMDA data set does. In fact, the RLSMDA data set stores only 78 and 34 miRNAs associated with breast cancer and prostate cancer respectively. However, the latest version of the three databases stores 227 and 152 miRNAs which have been found related to breast and prostate cancer. Thus, our predicted results can be fairly verified by the literature ground truth. As some of the predicted disease-miRNA associations were not covered by the three databases, we also searched other web sources to confirm the prediction results.

**Fig. 5:**
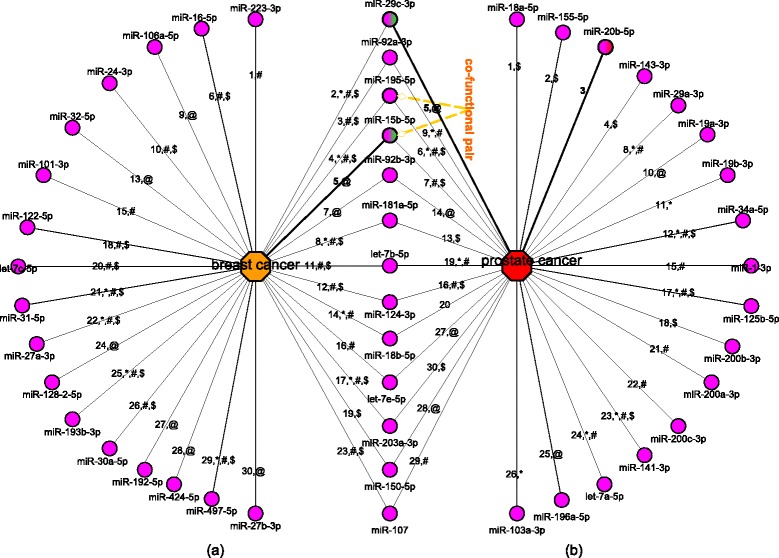
The top 30 predicted breast cancer-miRNA and prostate cancer-miRNA associations and the verification resources. The part **a** shows the predicted breast cancer related miRNAs and the part **b** gives the predict prostate cancer related miRNAs. The labels of the edges illustrate the rank of the predicted associations and the confirming types. The characters “*”, “#” or “$” stand for that the corresponding associations can be confirmed by the records in miR2Disease, HMDD or miRCancer respectively. The character “@” means that the association can be confirmed by other articles. A co-functional pair miR-195-5p-miR-15b-5p is highlighted

We constructed 100 prediction models (for making reliable predictions), each time using all the 1184 disease-miRNA pairs as the positive samples and a set of randomly selected 1184 negative samples from the negative_expression data set (a data set of 4638 negative samples based on the analysis of expression data). If a unknown cancer-miRNA relationship is positively predicted by all the 100 models, then a strong association exists between the cancer and the miRNA. The association probabilities derived by the 100 models is averaged to indicate the strong association. Figure 5 shows the 30 top-ranked positively predicted miRNAs related to breast and prostate cancer in terms of the average probabilities of the 100 models for the miRNAs. The edges at the (a) part represent the breast cancer-miRNA associations while the edges at the (b) part show the prostate cancer-miRNA associations. The labels on these edges represent the ranking positions and evidence type of the prediction results. The characters “*”, “#” or “$” stand for that the corresponding associations can be confirmed by the records in the miR2Disease database, the HMDD database or the miRCancer database respectively. The character “@” means that the association can be confirmed by other articles. Otherwise, the predicted associations could not be confirmed to our best knowledge. Overall, 58 of the 60 predicted disease-miRNA relationships can be verified by the newer databases or by other literature work.

**Fig. 6:**
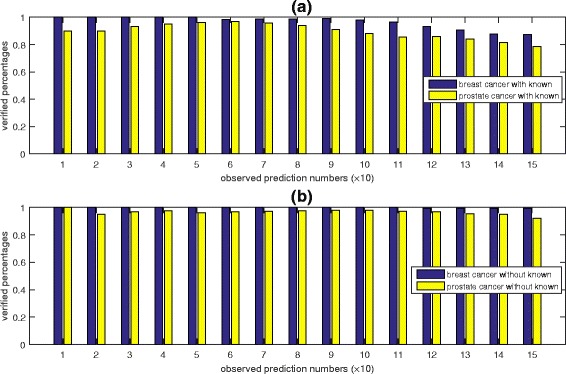
The percentages of the predicted disease-miRNA associations that can be verified. Panel **a** introduces the prediction performance of the model with the known cancer (breast and prostate cancer) related miRNAs. Panel **b** shows the prediction performance after the removal of the existing associations. The x-axis is the number of predictions (× 10) while the y-axis is the percentages of the verified predictions

Figure 6 a shows the percentages of the predicted disease-miRNA associations that can be verified when the number of top-ranked miRNAs varies from 10 to 150. The x-axis is the number of predictions (× 10) while the y-axis is the percentages of the verified predictions. For the first 10 to 50 predicted miRNAs associated with breast cancer or prostate cancer, 100 and 96% of them can be verified by the three newer databases or literature. The percentages drop to 98 and 88% when we assess on the first 100 predicted associations. This indicates that a more reliable predicted disease associated miRNAs can be ranked at a higher position by our method.

A novel association predicted by our method is about hsa-miR-15b (mapped as hsa-miR-15b-5p by miRBase) and breast cancer. Hsa-miR-15b is ranked as the 5th leading breast cancer related miRNA. This miRNA is an epidermal growth factor induced miRNA, and its association with breast cancer has not been recorded by any existing databases. However, a new discovery in 2015 can verify that there is an inverse correlation between the high expression of miRNA-15b and the low expression of its target gene MTSS1 in the tissues of breast cancer patients with the aggressive basal subtype [48]. The growth factor-inducible miRNAs can mediate the mechanisms underlying the progression of breast cancer. Another novel association predicted by our method is about hsa-miR-29c (mapped as hsa-miR-29c-3p by miRBase) and prostate cancer. This association is also ranked at the 5th position among the predicted prostate cancer related miRNAs, but it has not been recorded by any existing databases. A recent report claimed that miR-29c together with other five miRNAs such as miR-29a, miR-29b, miR-26a, miR-26b and miR-218 can control the expression of metastasis-promoting LOXL2 gene during the development of prostate cancer [49].

For the association miR-20b-prostate cancer which cannot be verified, Moltzahn et al. [50] had reported an upregulation of miR-20b in prostate cancer patients comparing with the healthy samples. However, this upregulation was not statistically significant at the follow-up PCR experiments. With our prediction, there may be an association between miR-20b and prostate cancer.

For some diseases, there have no currently known associations with any miRNAs. To test whether our prediction algorithm is still applicable for such situations, we conducted another experiment. In the experiment, we removed all the known miRNA associations with breast cancer or prostate cancer from the RLSMDA data set. The objective was to see whether our model can correctly predict these purposely removed and currently known breast cancer-miRNA or prostate cancer-miRNA associations. The prediction results are shown in Figure 6 b. Our model has a superior performance for predicting disease-miRNA associations even when there is no known association for these two cancers. Of the top 50 predicted disease-miRNA associations, all the predicted breast cancer-miRNA associations can be confirmed by the existing databases or literature, while 96% of the top 50 predicted prostate cancer-miRNA associations can be confirmed. The confirmation rates for the top-100 predicted associations can still maintain at a very high level. Moreover, the breast cancer-hsa-miR-15b-5p and the prostate cancer-hsa-miR-29c-3p can still be predicted and ranked highly. More details of the predicted and verified disease-miRNA pairs can be found in Additional file 2: Table S3–S6. The code in the Additional file 3 which implements our prediction algorithm has a default setting to output no more than 100 miRNAs for a given disease.

### Performance comparison: prediction of disease-miRNA relationships by different methods

A number of methods have been proposed to make predictions of unknown disease-miRNA relationships. We compared the performance of our prediction method with three state-of-the-art methods: RLSMDA [26], the method proposed by Xu et al. [28], and Jiang’s method [29]. RLSMDA is a semi-supervised method that does not need any negative samples. Xu’s method is a supervised approach and it collects the negative samples according to tissue-specific and expression properties of the miRNAs. Jiang’s method is also a supervised method. It has utilized a set of 270 negative samples randomly selected from the un-connected disease-miRNA pairs of a miRNA-disease bipartite network.

We first optimized our prediction model with two experiments such as selecting optimal precomputed kernel matrix and determining the best size of the negative samples comparing to positive ones during the training of our model. The results illustrate that our model can achieve best performance with the following settings: applying the squared root type of precomputed kernel matrix, setting the weight parameter *α*=0.8 and selecting the same size of negative samples as positive samples. In addition, we did the permutation test [29] which has proved that the performance of our prediction model was not produced occasionally but contains biological significance. The performances of our model with negative samples selected from our collected negative sample set or from all the un-connected pairs were compared. Selection of negative samples from our negative sample set has been proved to be a better choice. More detail of these procedures and the results can be found in the contents and Additional file 2: Figure S1–S3.

The source codes of these above three literature methods were not available. We implemented the RLSMDA algorithm, but not the complicated Xu’s or Jiang’s method. It is not possible for us to compare various methods with a independent test set. For a fair comparison, their data sets and performance metrics (specificity, recall(or sensitivity), precision, accuracy and AUC) were exactly used by our method. More details of the implementation and data sets are described in Additional file 2, the positive samples are listed in Additional file 4.

**Fig. 7:**
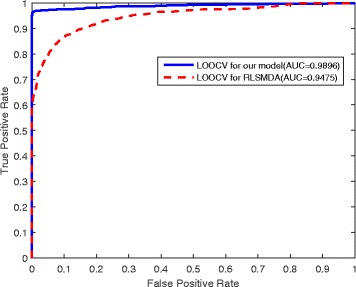
The ROC curves of our model compared with RLSMDA based on the same positive samples. The comparison is based on the same positive sample set and the different prediction model of RLSMDA and our newly designed model. The average AUC value of our model is 0.9896 while the RLSMDA obtains the lower value of 0.9475

The specificity, recall, accuracy and AUC performances are benchmarked in Table 2 (those values of Xu’s method and Jiang’s method were obtained from their published papers). The ROC curve of our method is depicted in Fig. 7 in comparison with the curve of the RLSMDA method under the same data set and the same leave-one-out cross-validation (LOOCV). The ROC curves for the comparison of our methods with all the three methods are also showed in Additional file 2: Figure S4–S6. Our prediction model achieves much better AUC values than the three state-of-the-art methods. This is the main reason why our prediction method was used to predict unknown miRNA-disease associations and the top-ranked ones were added to reconstruct the disease-gene-miRNA tripartite networks. The superior performance of our prediction method is mainly attributed to the careful selection of reliable negative samples as well as the precomputed kernel matrix which can identify more positive samples.

**Table 2:** Performance comparison between our method and the three state-of-the-art prediction methods

Methods	sample size	cv type	Specificity	Sensitivity	Accuracy	AUC
RLSMDA	1184+	LOOCV	–	–	–	0.9475
our model	1184+,1184-	LOOCV	0.9367	0.9368	0.9367	**0.9896**
Xu’s method	37+, 44-	5-fold	0.8833	0.8643	0.8772	0.9189
our model	37+, 37-	5-fold	**0.9990**	**1.000**	**0.9995**	**0.9854**
Jiang’s method	270+, 270-	10-fold	0.9125	0.7338	0.8232	0.8884
our model	263+, 263-	10-fold	**0.9274**	**0.8982**	**0.9128**	**0.9871**

Symbols “+/-” represent “positive samples/negative samples”. cv means cross-validation

The best performance among the compared methods are showed in boldface

## Discussion

A more challenging problem is to detect and prioritize multi-disease associated co-functional miRNA groups containing more than two members. An intuitive approach is to integrate all the co-functional pairs that overlap. As the co-function of more than two miRNAs is rarely reported by the literature, it is hard to validate the existence of multi-miRNA co-functional groups. The prioritization of multi-miRNA co-functional groups is one of our future research topics.

As demonstrated, our method has a much better performance for the prediction of disease-gene associations. A key idea of our method is the use of reliable negative samples screened and obtained through the fold change information of miRNA expressions. An alternative approach to the screening can be through pairwise test statistics. Combining expression fold change and pairwise test statistics may lead to decreasing the false positives in the set of negative samples. In the future work, we will examine the trade-off between the performance improvement and the quadratic pairwise time complexity.

Multi-disease associated co-functional miRNA pairs are also linked to the concept of competing endogenous RNAs (ceRNAs) [51, 52] which covers the regulation relationships of miRNAs and their targets including both mRNAs and long non-coding RNAs. Characteristics of miRNAs in the ceRNA networks can be considered to analyze the co-functions of miRNAs. For instance, if two miRNAs are involved in the same ceRNA network, they may always co-function with each other.

## Conclusion

We have conducted a cross disease analysis of co-functional miRNA pairs on a reconstructed disease-gene-miRNA tripartite network. We made the following contributions: (1) We proposed a new idea for selecting reliable negative samples of disease-miRNA relationship which can overcome the problem of lacking negative samples for machine learning methods to make reliable predictions of disease-associated miRNAs; (2) Our prediction model does not need to do feature selection, and it is applicable for large scale prediction of disease-associated miRNAs; (3) Our prediction model can work well for those miRNAs that have no currently known miRNA-disease associations; (4) We designed a scoring function to prioritize the candidate multi-disease associated co-functional miRNA pairs and their potential co-regulated genes; (5) We performed detailed case studies to understand the miRNA co-functional phenomenon for both cancers and non-cancer diseases; and (6) We performed deep case studies to reveal novel associations between miRNAs and breast cancer and those between miRNAs and prostate cancer. It can be concluded that our prediction method has a superior performance for the prediction of unknown miRNA-disease associations, and that the integration of the top-ranked ones into the existing database is useful and effective for the cross disease analysis of co-functional miRNA pairs.

## Methods

Our method for the detection and prioritization of co-functional miRNA pairs and cross disease analysis includes three main computational steps: (i) Reconstructing the DGR tripartite network by combining the known relationships of diseases, miRNAs, and genes with those predicted disease-miRNA associations, (ii) Ranking the candidate co-functional miRNA pairs via a novel scoring method, (iii) Determining the potential co-functional target genes of these co-functional miRNA pairs. Details of these steps and data sets are described in the following subsections.

### Data sets for the diseases, miRNAs and their related genes

Diseases and miRNAs stored at different databases may have different names or IDs. To deal with this inconsistency issue, we mapped the names of the diseases and miRNAs from all the relevant databases to the database Disease Ontology (DO) [53] and miRBase v21.0 [54]. The Medical Subject Headings (MeSH) [44] and Comparative Toxicogenomics Database (CTD) [55] were used as the dictionaries of the disease names. We searched in DO for all the disease names of a data set. When exact terms were found in DO, the names and the DO ids were recorded and stored in a separate file. Otherwise, we searched in MeSH and CTD, and used their synonyms to map them to DO terms. To map the names of the miRNAs, we searched the given ids of the miRNAs in miRBase v21. When a term was not found, then it was discarded (according to miRBase, it may be a dead record because it is not a miRNA, or the record has been replaced by another one). A miRNA id is always related to two mature miRNA ids with the suffix of ‘-5p’ or ‘-3p’ which means a precursor miRNA will generate two mature miRNAs from the 5’-arm or the 3’-arm respectively. As the mature miRNAs are the real functional parts, the miRNAs from different resources were mapped to the mature miRNA ids in miRBase v21. For those older version ids, we also mapped them to the current mature miRNA ids according to the term of Previous IDs of the miRBase database. Finally, each miRNA was mapped to one mature miRNA id of the database miRBase v21.

The genes were mapped to the entrez gene ids according to the HUGO Gene Nomenclature Committee (HGNC) [56]. To get the disease-related genes, we downloaded the supplementary files of [57] which contains 117,190 associations between 2817 diseases and 12063 genes from the database SIDD [58]. After data correction and redundancy removal, we obtained a data set of 114754 disease-gene associations between 2802 diseases and 10893 genes. To get the target genes of those miRNAs, we searched two databases: miRecords [19] and miRTarBase [20]. After mapping the miRNAs to miRBase v21 and mapping the gene names to entrez gene ids, we retrieved 322,269 miRNA-target pairs between 2588 miRNAs and 14794 genes. We list these disease genes and miRNA targets in Additional files 5 and 6 for more details.

### Positive samples and negative samples for training the prediction model to identify unknown disease-miRNA associations

There are several disease-miRNA databases such as miR2Disease [17], HMDD [18], and miRCancer [33]. This work focuses on the human mature miRNAs. The database HMDD stores the miRNAs as the precursor miRNA ids, these ids were first converted into mature miRNA ids according to the provided reference links before mapping them to the mature miRNA ids. After mapping the miRNAs and diseases to miRBase v21 and DO respectively, we retrieved 4578 associations between 463 miRNAs and 263 diseases from HMDD, 1952 associations between 83 cancers and 341 miRNAs from miRCancer, and 2096 disease-miRNA associations between 108 diseases and 287 miRNAs from miR2Disease. These are known disease-miRNA associations and they are used as the positive samples for the training of the prediction model.

Selection of negative samples, i.e., those disease-miRNA pairs that have little associations, is a difficult problem. We explored a novel idea to select credible negative samples. The new idea is to select negative samples according to the expression data of the miRNAs that we downloaded from the Gene Expression Omnibus (GEO) database [59]. We computed the fold changes of the miRNAs in the diseased patients comparing with the controls (i.e., the adjacent normal cells or the healthy contributor’s corresponding cells) according to the given platform information of the GEO database. A disease-related miRNA is always differentially expressed significantly between these two groups of subjects. Those miRNAs that are not significant differential expressed (the fold changes smaller than 0.05) will be regarded as non-disease related miRNAs. After conducting analysis on 78 GSE accessions (some accessions without enough information for compute the fold changes were removed), we determined 21432 disease-miRNA pairs between 2473 miRNAs and 73 diseases which have little association. The accession ids can be found in the Additional file 7. By comparing this data set of negative samples with the above HMDD-based, miRCancer-based and the miR2Disease-based positive data sets, those pairs that appeared in both of the negative data set and the positive data sets were discarded. We then obtained 4041, 1838 and 1487 disease-miRNA pairs respectively from HMDD,miRCancer and miR2Disease, which were regarded as positive samples. 20772 disease-miRNA pairs extracted by the analysis of the GSE accessions were used as negative samples. To obtain more reliable negative samples, we also removed those diseases that have no known related miRNAs and those miRNAs that have no known related diseases according to the three positive data sets from the 20772 disease-miRNA pairs. Finally, there are 4638 negative samples involving 53 diseases and 538 miRNAs. All these four data sets are further described in Additional file 8.

We note that Jiang’s method [29] takes all those unknown disease-miRNA pairs as negative samples and constructed balanced data sets by a random selection of a subset of the negative samples as the same size of the verified disease-miRNA associations. Xu’s method [28] takes those miRNAs at the lowest expression levels in the normal tissue as negative samples. Our method for selecting negative samples is different and more convincing as we consider the fold changes of the expression levels of the miRNAs between diseased and control tissues.

### Precomputed kernel matrices for the support vector machine

We applied support vector machine (SVM) to predict disease-related miRNAs. SVM is a supervised learning model for classification and regression [60]. We adopted the LibSVM version 3.20 [61] in this work. Usually, one can extract the features of the samples as the input of SVM to implement classification or regression with different kernel functions such as linear kernel, polynomial kernel, radial basis function kernel. However, even though we can represent a miRNA as a feature vector, it is hard to design an appropriate feature vector to describe a disease. Diseases are always phenotypes of patients. It is difficult to find the common properties of diseases that can be normalized as mathematical variables. To overcome this issue, we proposed to use precomputed kernel matrices instead of constructing the feature vectors to represent the disease-miRNA pairs. Construction of a precomputed kernel matrix has three main steps:

**Step 1**: Calculate the difference between two disease-miRNA pairs. Given two disease-miRNA pairs *d*_1_ *m*_1_ and *d*_2_ *m*_2_, we compute their difference (*d* *i* *f* *f*(*d*_1_ *m*_1_,*d*_2_ *m*_2_)) in three ways:

- Average approach:
  1 $$ \begin{aligned} diff\left(d_{1}m_{1},d_{2}m_{2} \right)=\left(DisSim\left(d_{1},d_{2} \right)+MiRSim\left(m_{1},m_{2} \right) \right)/2  \end{aligned}  $$
- Squared root approach:
  2 $$ \begin{aligned} diff\left(d_{1}m_{1},d_{2}m_{2} \right)=\sqrt{\left(DisSim\left(d_{1},d_{2} \right)\times MiRSim\left(m_{1},m_{2} \right) \right)}  \end{aligned}  $$
- Centre distance approach:
  3 $$ \begin{aligned} {}diff\!\left(d_{1}m_{1},d_{2}m_{2} \right)\!=&[ \left(DisSim\!\left(d_{1},d_{2} \right)\,-\,AvgDisSim \right)^{2}+\\ &\left(MiRSim\left(m_{1},m_{2} \right)\,-\,AvgMiRSim \right)^{2} \!]^{1/2}  \end{aligned}  $$

where *DisSim* and *MiRSim* represent the similarities between diseases and miRNAs respectively. *AvgDisSim* is the average similarity of all the disease-disease pairs, and *AvgMiRSim* is the average similarity of all the miRNA-miRNA pairs. Obviously, bigger values of *d* *i* *f* *f*(*d*_1_ *m*_1_,*d*_2_ *m*_2_) means the two pairs *d*_1_ *m*_1_,*d*_2_ *m*_2_ are more similar. Details of computing the similarities between diseases or between miRNAs are introduced in the next section.

**Step 2**: Constructing the kernel matrix for training samples. For a training set of *M* samples {*d*_1_ *m*_1_,*d*_2_ *m*_2_,…,*d*_*M*_ *m*_*M*_} with class labels {*l*_1_,*l*_2_,…,*l*_*M*_}, the training kernel matrix, denoted as *TKM*, is given by:

4 $$ TKM=\left(\begin{array}{ccc} k_{11} & \cdots & k_{1M}\\ \vdots & \ddots & \vdots\\ k_{M1} & \cdots & k_{MM} \end{array}\right)   $$

where, *k*_*ij*_=*diff*(*d*_*i*_ *m*_*i*_,*d*_*j*_ *m*_*j*_) is the difference between the two pairs *d*_*i*_ *m*_*i*_ and *d*_*j*_ *m*_*j*_.

**Step 3**: Constructing the kernel matrix for testing samples. For a testing set of *n* samples {*D*_1_ *M*_1_,*D*_2_ *M*_2_,…,*D*_*n*_ *M*_*n*_}, the kernel matrix for the testing samples, denoted by *PKM*, is given by:

5 $$ PKM=\left(\begin{array}{ccc} k^{\prime}_{11} & \cdots & k^{\prime}_{1M}\\ \vdots & \ddots & \vdots\\ k^{\prime}_{n1} & \cdots & k^{\prime}_{nM} \end{array}\right)   $$

Using *TKM* and *PKM* as input to libSVM, the class labels of the *n* testing samples can be predicted, and the probabilities of the predictions can be derived at the same time.

### Measuring the pairwise similarities of diseases or miRNAs

Disease similarity between two diseases, denoted by *DisSim*, is measured in two parts: the disease semantic similarity (*SemSim*) and the functional similarity between disease-related gene sets (*FunSim*). The multiplication of *SemSim* and *FunSim* is defined as *DisSim*. The definition of *FunSim* is referred to the SemFunSim method [57]. We implemented the algorithm and obtained the *FunSim* measurements between 2802 diseases. The *SemSim* was computed with the R package DOSE [62]. For the DOSE, we applied Resnik’s [63] definition of the common ancestor for two given terms. To avoid too many zero values of the similarities, we integrated *SemSim* and *FunSim* using a sum (instead of multiplication) and a weight parameter *α*. The new similarity measurement between disease *d*_*i*_ and disease *d*_*j*_ is computed by

6 $$ \begin{aligned} DisSim\left(d_{i},d_{j} \right)=\alpha\times FunSim\left(d_{i},d_{j} \right)+\left(1-\alpha \right)\times SemSim\left(d_{i},d_{j} \right)  \end{aligned}  $$

MiRNA similarity between two miRNAs, denoted by *MiRSim* is also measured in two parts: the sequence similarity (*SeqSim*) and the function similarity (*funSim*). *SeqSim* evaluates the similarity of the two miRNA sequences. We applied the idea of pseudo amino acid composition [64] to represent a miRNA as a (4+*λ*)-dimension vector. This idea was originally proposed to represent protein sequences as vectors.

Given a RNA sequence *R*:*r*_1_,*r*_2_,…*r*_*i*_,…*r*_*L*_, where *r*_*i*_∈{*A*,*G*,*C*,*U*}. Then, *R* is represented as a vector *V*_*R*_=[*v*_*i*_]_1×(4+*λ*)_, where the first four components stand for the occurrence frequencies of the 4 native nucleotides, and the latter *λ* components represent the sequence order effects of the nucleotides of *R*. The *t*-th (*t*<*L*) tier sequence order effect *θ*_*t*_ is calculated by

7 $$ \theta_{t}=\frac{1}{L-t}\sum_{i=1}^{L-t}\Theta\left(r_{i},r_{i+t} \right)   $$

8 $$ \Theta\left(r_{i},r_{i+t} \right)=\left(M_{i}-M_{i+t} \right)^{2}   $$

9 $$ M_{i}=\frac{M^{0}_{i}-\sum_{j=1}^{4}\frac{M^{0}_{j}}{4}}{\sqrt{\frac{\sum_{j=1}^{4}\left(M^{0}_{i}-\sum_{j=1}^{4}\frac{M^{0}_{j}}{4} \right)^{2}}{4}}}   $$

where, *M*_*i*_ is the normalized *i*th (*i*=1, 2, 3, 4) molecular weight of the nucleotide. The original molecular weights ($\textit {M}^{0}_{\mathrm {i}}$) of the four nucleotides are 135.1270 for A, 151.1261 for G, 111.1020 for C and 112.0868 for U. Then *V*_*R*_=[*v*_1_,*v*_2_,…*v*_*u*_,…,*v*_4+*λ*_],

10 $$ v_{u}=\left\{\frac{f_{u}}{\sum_{i=1}^{4}f_{i}+w\sum_{j=1}^{\lambda}\theta_{j}},\left(1\leq u \leq 4 \right) \atop \frac{w\theta_{u-4}}{\sum_{i=1}^{4}f_{i}+w\sum_{j=1}^{\lambda}\theta_{j}},\left(5\leq u \leq 4+\lambda \right)\right.   $$

In this work, we set *λ*=5 and the weight factor *w*=0.05. *f*_*u*_ is the occurrence frequencies of the nucleotide *u*. Then, each of the miRNA sequence *R* is represented as a 9-dimension vector *V*_*R*_=[*v*_*i*_]_1×9_. Overall, the sequence similarity is given by

11 $$ SeqSim\left(R_{i},R_{j} \right)=1-\frac{SeqDis\left(V_{i},V_{j} \right)-min(SeqDis)}{max(SeqDis)-min(SeqDis)}   $$

12 $$ SeqDis\left(V_{i},V_{j} \right)=\left| V_{i}-V_{j} \right|   $$

where, |·| is the Euclidean distance, and *min(SeqDis)* and *max(SeqDis)* represent the maximum value and the minimum value of all the *SeqSim* values between different miRNAs.

The *funSim* measurement is computed similarly as computing *FunSim*, namely a *funSim* between two miRNAs can be represented as the similarity between the two miRNA target sets. Similar to the measurement of *DisSim*, *MiRSim* of two miRNAs *R*_*i*_ and *R*_*j*_ is measured by integrating *funSim* and *SeqSim* with the same parameter *α* as follows:

13 $$ \begin{aligned} {}MiRSim\left(R_{i},R_{j} \right) &=\alpha\times funSim\left(R_{i},R_{j} \right)\\ &\quad+\left(1-\alpha \right)\times SeqSim\left(R_{i},R_{j} \right)  \end{aligned}  $$

Among all the datasets we mentioned previously, 551 different mature miRNAs were involved. Thus, we obtained the similarities between these 551 miRNAs in this work (details of the miRNAs and their targets listed in Additional file 6). Together with the similarities between 2802 diseases (details of the disease-gene associations listed in Additional file 5), these plenty of similarity information provide us adequate data to investigate associations between diseases and miRNAs.

### Scoring the multi-disease associated co-functional miRNA pairs

This work defines a multi-disease associated co-functional miRNA pair as a pair of miRNAs that can dysregulate the same gene or whose target genes are involved in the same cellular processes to participate in the development of a series of diseases. Such a miRNA pair has three good properties: (i) the members function cooperatively, which means they prefer to share the same targets; (ii) the members are associated with the development of a same set of diseases; and (iii) the common miRNA targets of the two miRNAs are potentially to be the common disease genes of their related diseases. These three properties can be examined on a DGR tripartite network containing various associations between miRNAs, diseases and genes.

Let *d* *g* *r*=(*V*_*d*_∪*V*_*g*_∪*V*_*r*_,*E*) be a DGR tripartite network, where *V*_*d*_ is a set of diseases, *V*_*g*_ is a set of disease genes, *V*_*r*_ is a set of disease-related miRNAs, and *E* is the associations between these diseases, genes, and miRNAs. Given a pair of miRNAs *R*_1_ and *R*_2_,*R*_1_,*R*_2_∈*V*_*r*_, we find the gene sets *G*_1_={*g*_11_,*g*_12_,…,*g*_1*k*_,…,*g*_1*m*_} and *G*_2_={*g*_21_,*g*_22_,…,*g*_2*t*_,…,*g*_2*n*_}, where *g*_1*k*_,*g*_2*t*_∈*V*_*g*_ and the edges (*R*_1_,*g*_1*k*_),(*R*_2_,*g*_2*t*_)∈*E*. We also find two subsets of diseases *D*_1_={*d*_11_,*d*_12_,…,*d*_1*p*_,…,*d*_1*x*_} and *D*_2_={*d*_21_,*d*_22_,…,*d*_2*q*_,…,*d*_1*y*_}, such that *d*_1*p*_,*d*_2*q*_∈*V*_*d*_ and the edges (*R*_1_,*d*_1*p*_),(*R*_2_,*d*_2*q*_)∈*E*. Then, for each disease *d*_*l*_ in *D*_1_ and *D*_2_, we can get its related genes $\textit {d}^{{g}}_{{l}}=\left \{\textit {g}_{1},\textit {g}_{2},\dots, g_{l}, \dots, \textit {g}_{z}\right \}$.

We quantify (i) the function relationship between a pair of miRNAs, (ii) miRNA regulation relationship in different diseases, and (iii) the relationship between the shared targets of two miRNAs and the common disease genes of these miRNAs associated diseases:

- MiRNA function relationship. A function relationship between *R*_1_ and *R*_2_ is quantified as the proportion of the shared targets (*p* *s* *g*(*R*_1_,*R*_2_)), namely,
  14 $$ psg\left(R_{1},R_{2}\right)=\frac{G_{1}\cap G_{2}}{G_{1}\cup G_{2}}   $$
- MiRNA regulation relationship in different diseases. The idea is that those miRNAs that have significant differential expression levels in different disease are more likely to function cooperatively. The co-dysexpression rate of *R*_1_ and *R*_2_,*r* *d*(*R*_1_,*R*_2_), is defined with consideration of their shared diseases and the percentage of the shared diseases comparing with all the diseases in *dgr* (i.e., |*V*_*d*_|):
  15 $$ rd(R_{1},R_{2})=\frac{D_{1}\cap D_{2}}{D_{1}\cup D_{2}}\cdot \frac{D_{1}\cap D_{2}}{|V_{d}|}   $$
- The relationship between the shared targets of *R*_1_ and *R*_2_ and the common disease genes of *R*_1_ and *R*_2_ shared diseases is defined as *p* *s* *g* *c*(*R*_1_,*R*_2_). The idea is that those co-functional miRNAs always co-dysregulate the common disease genes to contribute to the disease development.
  16 $$ psgc\left(R_{1},R_{2}\right)=\frac{\bigcup_{l=1}^{s}\left(\left(G_{1}\cap G_{2}\right)\cap d_{l}^{g}\right)}{G_{1}\cap G_{2}}   $$

where *s* is the number of diseases that the R1 and R2 shared.

The score for weighting the probability of the pair *R*_1_ and *R*_2_ to be a multi-disease associated co-functional pair (*cfScore*) is defined as:

17 $$ cfScore\left(R_{1}, R_{2}\right) = psg\left(R_{1}, R_{2}\right) \cdot rd\left(R_{1}, R_{2}\right) \cdot psgc\left(R_{1}, R_{2}\right)  $$

MiRNA pairs related to bigger number of diseases are more likely to reflect the general regulation mechanism. Thus, a threshold is set to control the number of diseases that the pair is associated with. There is no reliable data set for us to select an optimal threshold, we just set the threshold to be 10. We can then rank all the candidate co-functional miRNA pairs according to their *cfScores*. A higher position indicates the pair is more likely to be a multi-disease associated co-functional miRNA pair.

Usually, the two members of a co-functional miRNA pair can share more than one common targets. However, only part of them are really dysregulated by the miRNA pair during the development of the diseases (called the co-functional targets of this co-functional miRNA pair). As all those miRNAs shared targets can be candidate co-functional target, a probability is estimated for the candidate co-functional targets to be the exact dysregulated genes during the diseases’ developments. The idea is that the candidate co-functional targets being the disease genes for more of the miRNA pair associated diseases are more likely to be the real ones. We calculate the probability of gene *g*_*i*_,*p*(*g*_*i*_), to be a co-functional target by:

18 $$ p\left(g_{i}\right)=\frac{C_{g_{i}\cap\left(D_{1}\cap D_{2} \right)}}{C_{\left(D_{1}\cap D2 \right)}}   $$

where $\textit {C}_{\textit {D}_{1} \cap \textit {D}_{2}}$ is the number of common diseases associated with miRNA *R*_1_ and *R*_2_, while $\textit {C}_{\textit {g}_{i}\cap \left (\textit {D}_{1}\cap \textit {D}_{2} \right)}$ is the number of diseases associated with gene *g*_*i*_.

## Additional files

Additional file 1 The disease-miRNA associations for constructing the DGR tripartite. We list the disease-miRNA associations for constructing the DGR tripartite here including the cancer related tripartite and the non-cancer disease associated tripartite.This file can also be downloaded from: https://drive.google.com/open?id=0B6lH3mKdA9CSTkg2OVBPS0ZfVnM. (XLS 689 kb)

Additional file 2 The supplementary results for our work. This file mainly introduces the supplementary results of our work such as the details of the supplementary files, the details of the model comparison, the case study results, our prioritized multi-disease associated co-function miRNA pairs, the supplementary codes and the related references.This file can also be downloaded from: https://drive.google.com/open?id=0B6lH3mKdA9CSTkg2OVBPS0ZfVnM.
• **Figure S1.** Performances of the predictions under different precomputed kernel matrix and alpha.
• **Figure S2.** The ROC curves of the permutation test.
• **Figure S3.** Performances of the prediction models with different size ratio of negative and positive samples.
• **Figure S4.** The ROC curves of our model compared with RLSMDA based on the same positive samples.
• **Figure S5.** The ROC curves of our model and Xu’s based on the same positive sample set and 5-fold cross validation.
• **Figure S6.** The ROC curves of our method and Jiang’s method based on their positive sample set.
(PDF 1260 kb)

Additional file 3 Supplementary codes and data. The matlab codes of our methods and the input datasets. The data can also be downloaded from the following website: https://drive.google.com/open?id=0B6lH3mKdA9CSWDJHaWpnSUlPbGc. (ZIP 3080 kb)

Additional file 4 Datasets for the comparison of different miRNA-disease association prediction models. This file contains the three datasets that used in three state-of-the-art methods such as RLSMDA, Xu’s method and Jiang’s method.This file can also be downloaded from: https://drive.google.com/open?id=0B6lH3mKdA9CSTkg2OVBPS0ZfVnM. (XLS 157 kb)

Additional file 5 The disease-gene associations. The disease genes are listed in this file. These disease genes were obtained from the reference [56] and curated based on the DOID database and the HGNC database. This file can also be downloaded from: https://drive.google.com/open?id=0B6lH3mKdA9CSTkg2OVBPS0ZfVnM. (XLS 7220 kb)

Additional file 6 The miRNA-target associations. The miRNA targets were downloaded from the two databased such as the miRecords an miRTarBase. The miRNA ids were mapped according to the miRBase v21, while the genes were mapped to HGNC database records. This file can also be downloaded from: https://drive.google.com/open?id=0B6lH3mKdA9CSTkg2OVBPS0ZfVnM. (XLS 5320 kb)

Additional file 7 The GSE accessions for extracting negative samples. There are totally 78 GSE accessions that we downloaded from the GEO database. We analyzed these files to compute the fold changes of the miRNAs according to the given platform information.This file can also be downloaded from: https://drive.google.com/open?id=0B6lH3mKdA9CSTkg2OVBPS0ZfVnM. (XLS 28 kb)

Additional file 8 Datasets for constructing the miRNA-disease association prediction models. Four datasets such as three positive sample sets “positive_miR”, “positive_HMDD” and “positive_miRcancer” and the negative sample set “negative_expression” are stored. The three positive sample sets are retrieved from the three existing databases such as miR2Disease, HMDD v2 and miRCancer, while the negative sample set was obtained via analyzing the expression of the miRNAs.This file can also be downloaded from: https://drive.google.com/open?id=0B6lH3mKdA9CSTkg2OVBPS0ZfVnM. (XLS 947 kb)

## Abbreviations

AUC: Area under the ROC curve
CTD: Comparative toxicogenomics database
DGR: Disease-gene-microRNA
DO: Disease ontology
GEO: Gene expression omnibus
KMT: Kernel matrix type
LOOCV: Leave-one-out cross-validation
MeSH: Medical subject headings
microRNA: miRNA
OMIM: Online mendelian inheritance in man
SVM: support vector machine
