# Supplementary material for: Cross disease analysis of co-functional microRNA pairs on a reconstructed network of disease-gene-microRNA tripartite
Source: BMC Bioinformatics. 2017 Mar 24;18:193. doi: 10.1186/s12859-017-1605-0 (PMC5366146; doi:10.1186/s12859-017-1605-0)
Supplement: Supplementary file 2 — The supplementary results for our work. This file mainly introduces the supplementary results of our work such as the details of the supplementary files, the details of the model comparison, the case study results, our prioritized multi-disease associated co-function miRNA pairs, the supplementary codes and the related references.This file can also be downloaded from: https://drive.google.com/open?id=0B6lH3mKdA9CSTkg2OVBPS0ZfVnM. • Figure S1. Performances of the predictions under different precomputed kernel matrix and alpha. • Figure S2. The ROC curves of the permutation test. • Figure S3. Performances of the prediction models with different size ratio of negative and positive samples. • Figure S4. The ROC curves of our model compared with RLSMDA based on the same positive samples. • Figure S5. The ROC curves of our model and Xu’s based on the same positive sample set and 5-fold cross validation. • Figure S6. The ROC curves of our method and Jiang’s method based on their positive sample set. (PDF 1260 kb) [file 12859_2017_1605_MOESM2_ESM.pdf]

Cross disease analysis of co-functional miRNA pairs on a big reconstructed network of disease-gene-miRNA tripartite

Hui Peng[1], Chaowang Lan[1], Yi Zheng[1], Gyorgy Hutvagner[2] Dacheng Tao[3], Jinyan Li[1,*]

1. Advanced Analytics Institute, University of Technology Sydney, PO Box 123, Broadway, NSW 2007, Australia.

2. Centre for Health Technologies, University of Technology Sydney, PO Box 123, Broadway, NSW 2007, Australia.

3. School of Information Technologies and the Faculty of Engineering and Information Technologies, University of Sydney, J12/318 Cleveland St, Darlington NSW 2008， Australia.

*** Corresponding Author**

E-mail: Jinyan.Li@uts.edu.au

1. Supplementary information introduction

There are totally 8 supplementary files in Supplementary information. The **Supplementary file 1** lists the disease-miRNA associations that we used to construct the DGR tripartite networks. **Supplementary file 2** mainly introduces the details of optimizing our disease-miRNA association prediction models and the comparison of it with the other existing methods. There are several Supplementary Tables which list the results of the case studies of the disease-miRNA association prediction model, the top 50 candidate multi-disease associated co-functional miRNA pairs for cancer related DGR tripartite network and the non-cancer disease related DGR tripartite network. In **Supplementary file 3**, we provide the related codes and datasets of our methods. As the data files of the similarities between 2802 diseases and the similarities between 551 miRNAs are too big, if someone need these datasets, please contact Hui Peng (email: hui.peng-2@student.uts.edu.au). **Supplementary file 4** shows the three data sets that applied by chen's method, xu's method and jiang's method, which we used them to make comparison with these three existing methods. In **Supplementary file 5 and Supplementary file 6**, we list the disease-gene associations and miRNA targets that we adopted to compute the disease similarities, miRNA similarities and find co-function miRNA pairs. **Supplementary file 7** contains the GSE accession ids from the GEO database, which we used to obtain the reliable negative disease-miRNA association samples. The final samples of disease-miRNA associations from different databases are listed in **Supplementary file 8** including the mapped disease DO ids, mature miRNA ids, the negative sample set according to the analysis of the miRNA expression level fold changes, and four positive sample sets.

The five matlab code files such as ComKerMat.m, PreDisRNA.m, DMMD.m, CoFunScore.m and CoFunScoreAll.m are the codes that implemented our methods. We paste all the codes at the 5th section of this supplementary file. The first two code files are used for predicting disease-miRNA associations while the later three code files are for prioritizing the multi-disease associated co-functional miRNA pairs. The input data can be found from the Supplementary files. The node in

the code files have illustrated the meaning of the inputs, outputs and data structures. For prediction of disease-miRNA associations, the PreDisRNA.m is the main interface. The CoFunScoreAll.m is the main interface for prioritizing the multi-disease associated co-functional miRNA pairs.

2. The optimal precomputed kernel matrix and the prediction performance

During the optimization of our method and the comparison of different prediction methods, the following seven performance metrics were computed: specificity, recall (or sensitive), precision, accuracy, F1 and AUC (area under the ROC curve). The definition of mcc is given by:

$$\mathrm{mcc} = \frac{TP \times TN - FP \times FN}{\sqrt{(TP + FP)(TP + FN)(TN + FP)(TN + FN)}}$$

where *TP*, *TN*, *FP* and *FN* represent true positive, true negative, false positive and false negative respectively.

There are a weight parameter α and a kernel matrix type *KMT* which can be properly set to build an optimal prediction model in this work. Parameter α is used to mediate the similarities between diseases and the similarities between miRNAs, while *KMT* selects a kernel matrix type for support vector machine (SVM) to make accurate classification. Detailed explanation of and *KMT* can be found in **Methods**. Experiments for the proper selection of and *KMT* were conducted under three steps: (1) construction of training data. We extracted 1487 known disease-miRNA associations between 107 diseases and 276 miRNAs from the miR2Disease database, and used them as the set of positive training samples (denoted as positive_miR). We also constructed a set of 4638 negative samples between 53 diseases and 538 miRNAs after a comprehensive analysis of the GSE accessions (denoted as negative_expression). We randomly selected 1487 negative samples from negative_expression to construct a balanced training data set; (2) prediction model construction. This step has two layers of loops. The outer loop changes the value of α from 0 to 1 with a step of 0.1, while the inner loop sets *KMT* = 1, 2, or 3, which represent the three different types of kernel matrices (i.e., the average type, the squared root type and the center distance type). A prediction model was constructed with each α and *KMT*; (3) performance evaluation. We implemented 10-fold cross-validation on the balanced data set with different α and *KMT* and the seven performance metrics (Specificity, Recall, Precision, Accuracy, F1, mcc and AUC) were computed. We ran the experiment 100 times. The averages of the seven indices were taken over the 100 times. **Supp. Fig. 1**. shows the AUC values and F1 scores.

The squared root type of KMT outperforms the other two types. When α increases, the AUC and F1 score increase first but then drop down, suggesting that the integration of different types of similarities can improve the prediction performance. Furthermore, when $\alpha = 0$ or $\alpha = 1$, the average type and the squared root type can still achieve the AUC values around 0.92 and F1 scores about 0.9. It means that our precomputed kernel matrix method can have a good prediction performance even with just one kind of similarity information. Comparing the curves in **Supp. Fig. 1**, it can be seen when α is around 0.8, the curves achieve better AUCs and F1 scores. Thus, we chose the squared root type of *KMT* and set $\alpha = 0.8$ for our prediction model.

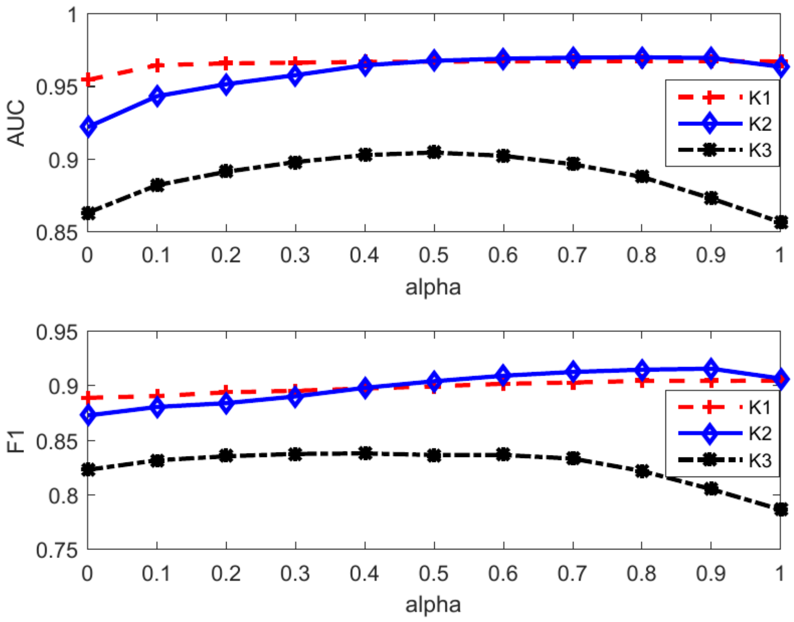

Supp. Fig. 1. **Performances of the predictions under different precomputed kernel matrix and alpha.** We mainly compare the AUC values and the F1 scores of each model with different parameters. K1, K2 and K3 represent the three kernel matrix types such as the average type, the squared root type and the center distance type respectively. The results indicate that the model with the squared root type of kernel matrix and alpha=0.8 achieves better performance.

To evaluate whether our prediction performance was obtained by chance, we conducted a permutation test as Jiang et al. [3] did. We did not use the true labels of the samples (positive samples and negative samples) but distributed the labels randomly. Then, we implemented the 10-fold cross-validation and observed the changes of the performance. The positive_miR data set was adopted as the positive samples and balanced training data sets were built. The normal predictions (true labels) were considered as the control group while the permutation tests were regarded as the test group. All these two groups of experiments were repeated 10 times. The ROC curves of the test group and control group are shown in **Supp. Fig. 2**. The ROC curve of the test group is nearly overlapped with the random lines while the ROC curve of the control group can achieve an AUC value of 0.97, which indicates that the performance of our prediction model was not produced occasionally but contains biological significance.

3. Effect of the size of the negative samples on the prediction performance

To investigate whether the number of negative samples affects the performance of our predictions, we fixed the size of positive samples as the size of the positive_miR data set, and changed the number of negative samples in the training data set. All the negative samples were randomly selected from the negative_expression data set. We varied the number of negative samples from 3 times the number of positive samples to 2 times, to equal size, and to 80% of the size of positive samples, 60%, 40% and 20%. In addition, the positive samples from the positive_HMDD (totally 4041positive samples which were extracted from the HMDD database) excluding those samples already in the data set of positive_miR were adopted to build the validation data set. There are 3484 positive samples in this validation data set. Again, 10-fold cross-validation was implemented on the training data. The prediction model was then tested on the

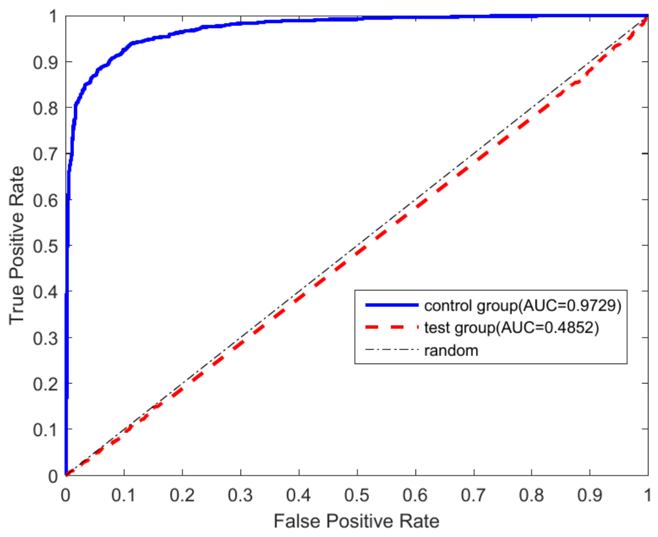

Supp. Fig. 2 The ROC curves of the permutation test. The experiment includes the test group and the control group parts. The test group part used the permutated labels for the training samples while the control group part uses the original labels of the same training dataset. Both two parts of the experiment adopts our optimal prediction model.

validation data set. As the samples in the validation data set are all positive samples, we just computed the accuracy but not other metrics. All the experiments were repeated 100 times. The average performances are depicted in **Supp. Fig. 3** to show the changes of AUC and mcc values of the cross-validation experiments and the accuracy based on the validation dataset when the size of negative samples changes (the size ratio between the negative and positive samples is displayed on the x-axis).

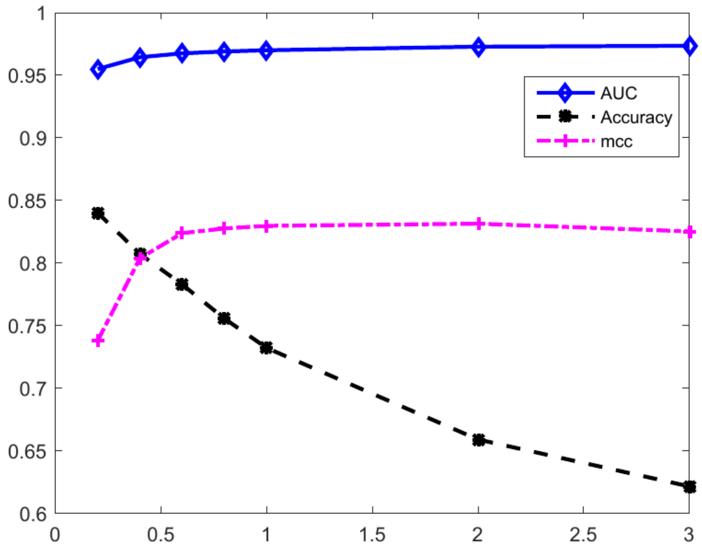

Supp. Fig. 3 Performances of the prediction models with different size ratio of negative and positive samples. The prediction model was trained on the sample sets with different ratio of negative and positive samples. The x-axis shows the ratios. AUC and mcc values were computed based on 10-fold cross validation. The Accuracy is the percentage that the samples in the validation dataset (a dataset with just positive samples but does not overlap with the training sample sets) are predicted correctly.

We can find that the AUC values have nearly no changes under different size ratios between negative and positive samples. However, the accuracy of the prediction on the validation data set drops when the size of negative samples increases. But, the mcc value increases till the size of negative samples is equal to that of positive samples. Then, it keeps at the same level even more negative samples are added. As mcc is a more comprehensive performance index than accuracy, we suggest that a balanced training data set of positive and negative samples should be adopted to infer new disease-miRNA associations as we did in this work.

4. Performance comparison when changing the approach of selecting negative samples

The negative samples of disease-miRNA relationship randomly selected from the negative expression data set were used by this work for the training of the prediction model. There are other ways for the construction of negative data sets, such as by random selection from the unconnected disease-miRNA pairs. We compared the performances of our prediction model when the approach to select negative samples was changed. The positive samples were always the same, i.e., the data set positive_miR containing 1487 known disease-miRNA associations.

The negative data set formed by a random selection from those unconnected disease-miRNA pairs is named negative_random (there are total 26704 disease-miRNA pairs). We conducted two experiments. In the first experiment, we used all the 1487 positive samples from positive_miR and 1487 negative samples randomly selected from the negative_expression data set to build the training data set. The second experiment is similar to the first one with the only difference that the 1487 negative samples were randomly selected from negative_random. 10-fold cross-validation was conducted on the training data sets. To get a test performance, we also used the above validation data set to test the prediction models. All these experiments were repeated 100 times, and the average performance was taken to reduce the bias of the predictions (**Supp. Tab. 1**).

Supp. Tab. 1 The prediction performances based on different approaches to select negative samples

**negative**	**10-fold cross validation**							
	specificity	recall	precision	accuracy	F1	mcc	AUC	Accuracy
**expression**	0.9194	0.9107	0.9191	0.9151	0.9147	0.8306	0.9704	0.7315
**random**	0.7719	0.7808	0.7746	0.7764	0.7773	0.5534	0.7315	0.5077

It is clear that the 10-fold cross-validation performance of selecting negative samples from negative_expression significantly outperformed another approach. For the 3484 samples of the validation data set, 73.15% of them can be correctly predicted by the model when the negative samples were selected from negative_expression, while the negative_random based model could only accurately predict 50.77% of the 3484 disease-miRNA associations. This comparison indicates that the approach for the selection of negative samples has significant impact on the prediction performance. The best choice is to select negative samples based on the analysis of expression data.

5. Comparing with other's methods

As several methods have been proposed to infer the disease-miRNA relationships, it's necessary for us to make comparison of our prediction model with those existing methods. Here we choose the representative non-machine learning method based and two machine learning method based prediction models for the

comparisons. The first model is the RLSMDA that proposed by Chen et al. [1]. As the author didn't provide the source code of their prediction model, we adopted the data they provided and the algorithm they introduced in their paper, and implemented their model. Chen et al. reported that they had done the local leave-one-out cross validation (local LOOCV) and global leave-one-out cross validation (global LOOCV) on their dataset with 1395 known disease-miRNA associations, including 271 miRNAs and 137 diseases. We mapped the diseases and miRNAs to DO and miRBase v21, and finally obtained 1184 disease-miRNA pairs. We also mapped these 1184 disease-miRNA pairs to our data set. We implemented the global LOOCV on RLSMDA and our prediction method. We randomly selected 1184 disease-miRNA pairs from negative_expression as the reliable negative samples. The LOOCV was repeated 10 times for our model so that we selected 10 different negative sample sets. The indices were the average values of those 10 runs. The ROC curves of our method and the RLSMDA is showed in **Supp. Fig. 4**.

According to **Supp. Fig. 4**, our prediction model can achieve better performance than the RLSMDA based on the same positive samples and the leave-one-out cross-validation (AUC value = 0.9896 for our model and AUC value=0.9475 for RLSMDA).

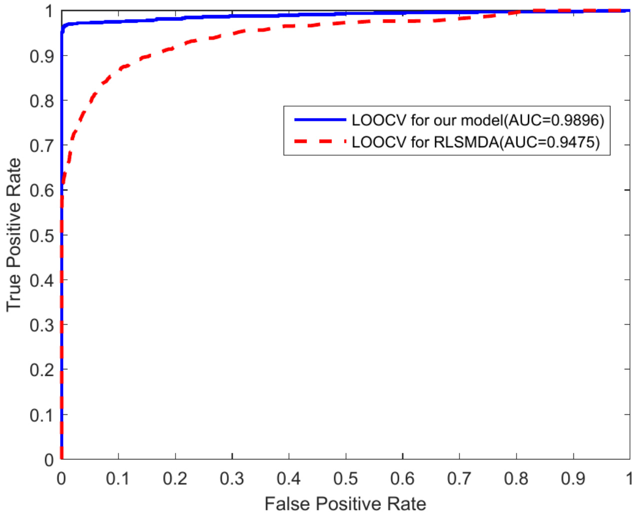

Supp. Fig. 4 The ROC curves of our model compared with RLSMDA based on the same positive samples

There are other two SVM based prediction models such as the method proposed by Xu et al. [2] and Jiang et al. [3]. Both two methods adopted the traditional idea that using the feature vectors of the disease-miRNA pairs as the input and then training and testing the samples. However, the feature vectors of Xu's method are hard to collect as they used the topological properties of the miRNA target–dysregulated network and the fold-change of the miRNA expression level. This method is hard to predict different kinds of diseases relate miRNAs simultaneously. They applied the 37 prostate cancer miRNAs as the positive samples and selected another 44 tissue-specific miRNAs with low expression levels as the negative samples. To compare with this method, we used these 37 prostate cancer miRNAs as the positive samples and randomly selected 37 disease-miRNAs from the negative-expression as the negative samples and then implemented the 5-fold cross-validation. As the sample size is small, we repeated the 5-fold cross-validation 1000 times, thus, we randomly selected 1000 negative sample sets, and the final evaluation indices are the average values of the 1000 runs. The ROC curves are listed in **Supp. Fig. 5**. The ROC curves of Xu's method are also showed in the right part of **Supp. Fig. 5** (the curves were obtained from the Ref. [2]).

According to the **Supp. Fig. 5**, based on the same positive samples and the 5-fold cross-validation, our model can achieve the AUC value of 0.9854 which is better than that of Xu's method of 0.9189. Though, this experiment is based on a small sample set with less than 100 samples and just a kind of disease, we can draw the conclusion that our model can achieve better performance on this special dataset. We didn't implement Xu's method, thus it's impossible for us to make comparisons of our model with Xu's based on more diseases.

To compare with Jiang's method in Ref. [3], we also downloaded the positive samples that they adopted to evaluate the performance of their model. There are 270 disease-miRNA pairs in this positive sample set.

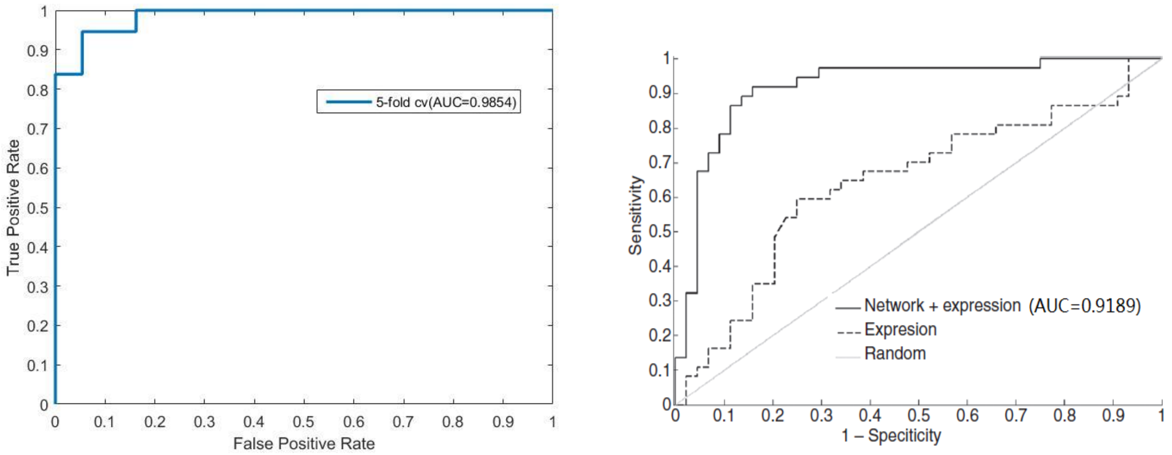

Supp. Fig. 5 The ROC curves of our model and Xu's based on the same positive sample set and 5-fold cross validation

After mapping the diseases to DO and mapping the miRNAs to miRBase v21, there are 263 disease-miRNA pairs that can finally mapped to our disease and miRNA sets. We adopted these 263 disease-miRNA pairs as the positive samples and randomly selected 263 negative samples from negative-expression as the gold standard data set. 10-fold cross-validation was implemented on this data set for 100 times. The ROC curve of our method and Jiang's (the curves were obtained from Ref. [3]) are showed in **Supp. Fig. 6**. The indices are listed in **Supp. Tab. 2** as comparison of the two prediction methods.

Supp. Tab. 2 The comparison of our method and Jiang's method in Ref. [3] based on their positive sample set with the 10-fold cross-validation

Method	**Recall**	**Specificity**	**Accuracy**	**AUC**
Our method	0.8982	0.9274	0.9128	0.9871
Jiang's PITA based	0.7338	0.9125	0.8232	0.8884

From **Supp. Tab. 2** and **Supp. Fig. 6**, we can find that based on the same positive sample set, all the evaluation index of our model is higher than that of Jiang's method. Furthermore, their negative samples were randomly selected from the disease-miRNA pairs that excluded the positive samples, which are not reliable enough in fact.

Above all, we can draw the conclusion that our method can achieve better performance than the existing methods for inferring the disease-miRNA relationships based on the cross-validations with different sample

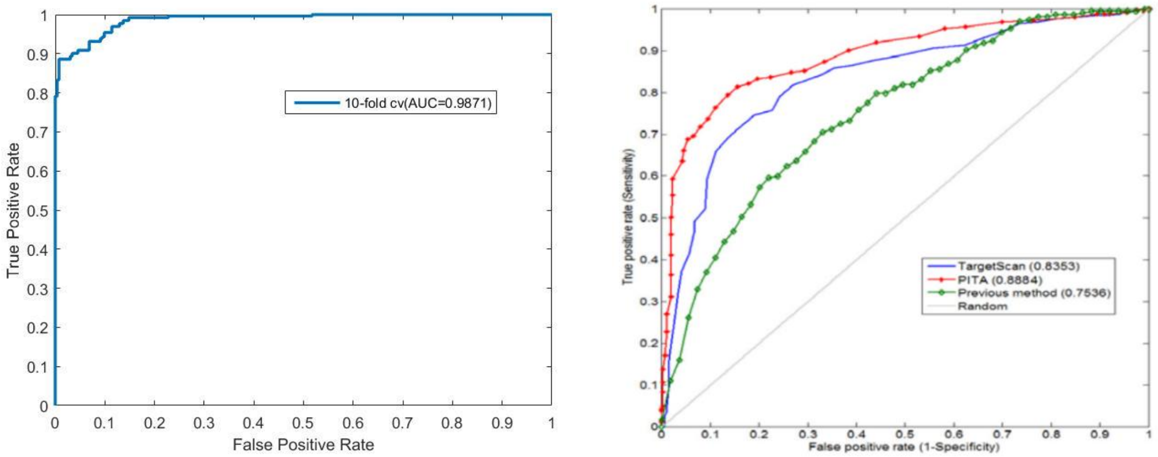

Supp. Fig. 6 The ROC curves of our method and Jiang's method based on their positive sample set. The right part of the figure was obtained from the corresponding published article.

sets. All the datasets for cross-validations can be found in the **Supplementary file 3**.

6. *The details of the predicted and confirmed predictions*

Supp. Tab. 3 The predict breast cancer-miRNAs with known breast cancer miRNA associations and the confirmation source

miRNA	possibility	evidence	miRNA	possibility	evidence	miRNA	possibility	evidence
**miR-223-3p**	0.9995	# [a]	miR-212-3p	0.9929	[13]	miR-520h	0.9594	#
**miR-29c-3p**	0.9994	* [c],#,$ [b]	miR-26b-5p	0.9929	#,$	miR-33b-5p	0.9572	[30]
**miR-92a-3p**	0.9994	#,$	miR-148a-3p	0.9926	*,#,$	miR-421	0.9520	[31]
**miR-195-5p**	0.9993	*,#,$	miR-130b-3p	0.9926	[14]	miR-216a-5p	0.9514	[24]
**miR-15b-5p**	0.9993	[4] [d]	miR-148b-3p	0.9923	#	miR-216b-5p	0.9514	$
**miR-16-5p**	0.9992	#,$	miR-95-5p	0.9923	[5]	miR-208a-3p	0.9473	
**miR-92b-3p**	0.9992	[5]	miR-133b	0.9923	#	miR-494-5p	0.9469	$
**miR-181a-5p**	0.9991	*,#,$	miR-96-5p	0.9920	*,#,$	miR-217	0.9457	$
**miR-106a-5p**	0.9990	[6]	miR-128-3p	0.9919	#,$	miR-598-5p	0.9450	
**miR-24-3p**	0.9989	#,$	miR-198	0.9914	[e]	miR-365a-3p	0.9442	*,#
**let-7b-5p**	0.9988	#,$	miR-363-3p	0.9912	[15]	miR-382-5p	0.9365	[32]
**miR-124-3p**	0.9988	#,$	miR-340-5p	0.9910	#	miR-154-5p	0.9365	
**miR-32-5p**	0.9987	[5]	miR-484	0.9908	[16]	miR-377-3p	0.9360	
**miR-18b-5p**	0.9987	*,#	miR-184	0.9902	[17]	miR-532-5p	0.9344	[33]
**miR-101-3p**	0.9983	#	miR-33a-5p	0.9900	[18]	miR-658	0.9339	
**let-7e-5p**	0.9982	$	miR-30e-5p	0.9898	[19]	miR-423-3p	0.9289	#,$
**miR-203a-3p**	0.9978	*,#,$	miR-135a-5p	0.9887	#,$	miR-152-5p	0.9284	#,$
**miR-122-5p**	0.9975	#,$	miR-197-3p	0.9882	#,$	miR-583	0.9278	
**miR-150-5p**	0.9974	$	miR-186-5p	0.9881	[5]	miR-136-5p	0.9266	*
**let-7c-5p**	0.9974	#,$	miR-211-5p	0.9878	$	miR-328-5p	0.9258	#
**miR-31-5p**	0.9974	*,#,$	miR-140-5p	0.9873	#,$	miR-371a-3p	0.9241	
**miR-27a-3p**	0.9973	*,#,$	miR-663a	0.9864	#	miR-431-5p	0.9206	

**miR-107**	0.9972	#,$	miR-615-3p	0.9863	[20]	miR-498	0.9205	[34]
**miR-128-2-5p**	0.9971	[7]	miR-130a-3p	0.9851	$	miR-337-3p	0.9178	[35]
**miR-193b-3p**	0.9968	*,#,$	miR-520a-3p	0.9838	#	miR-512-5p	0.9155	[36]
**miR-30a-5p**	0.9968	#,$	miR-449b-5p	0.9832	[21]	miR-208b-5p	0.9151	
**miR-192-5p**	0.9967	[5]	miR-520b	0.9827	#	miR-660-5p	0.9139	[37]
**miR-424-5p**	0.9966	[8]	miR-144-3p	0.9823	[22]	miR-455-5p	0.9072	
**miR-497-5p**	0.9966	*,#,$	miR-520c-3p	0.9811	#,$	miR-526b-5p	0.9057	$
**miR-27b-3p**	0.9964	[9]	miR-301b-3p	0.9808	#	miR-376c-3p	0.9015	#
**miR-98-5p**	0.9963	*,$	miR-520e	0.9801	[23]	miR-525-5p	0.9010	
**miR-126-3p**	0.9962	*,#,$	miR-342-3p	0.9801	#	miR-187-3p	0.9008	#
**miR-183-5p**	0.9961	#,$	miR-378a-5p	0.9801	$	miR-411-5p	0.8933	[38]
**let-7g-5p**	0.9956	#,$	miR-99b-5p	0.9797	[5]	miR-299-3p	0.8933	#
**miR-23b-3p**	0.9954	#	miR-301a-3p	0.9788	#	miR-652-3p	0.8878	[39]
**miR-181d-5p**	0.9954	*	miR-324-5p	0.9777	#	miR-561-3p	0.8878	
**miR-224-5p**	0.9952	#,$	miR-137	0.9770	#	miR-28-5p	0.8841	[5]
**miR-181c-5p**	0.9951	[10]	miR-330-3p	0.9761	[24]	miR-432-5p	0.8796	
**miR-375**	0.9949	[11]	miR-454-3p	0.9758	[25]	miR-514a-3p	0.8793	
**miR-100-5p**	0.9949	#,$	miR-139-5p	0.9749	#	miR-642a-5p	0.8756	
**miR-99a-5p**	0.9944	$	miR-185-5p	0.9739	$	miR-376a-3p	0.8733	[40]
**miR-22-3p**	0.9942	*,#,$	miR-372-5p	0.9738	[5]	miR-381-3p	0.8710	$
**miR-182-5p**	0.9941	*,#,$	miR-23a-3p	0.9737	#,$	miR-190a-5p	0.8678	[36]
**miR-191-5p**	0.9938	*,#,$	miR-331-3p	0.9734		miR-370-5p	0.8633	[41]
**miR-373-3p**	0.9938	*,#,$	miR-448	0.9730	[26]	miR-300	0.8546	$
**miR-449a**	0.9937	[12]	miR-433-5p	0.9663	[27]	miR-654-5p	0.8521	
**miR-142-5p**	0.9935	$	miR-129-5p	0.9657	#,$	miR-539-5p	0.8519	
**miR-199b-5p**	0.9932	#	miR-134-5p	0.9614	[28]	miR-486-5p	0.8457	[42]
**miR-26a-5p**	0.9931	*,#,$	miR-491-5p	0.9601	[29]	miR-608	0.8451	#
**miR-335-5p**	0.9930	*,#,$	miR-452-5p	0.9600	#	miR-557	0.8378	[43]

Note:

a: # represents this association has been confirmed by the HMDD database;

b: $ represents this association has been confirmed by the miRCancer database;

c: * represents this association has been confirmed by miR2Disease database.

d: [1] represents that this predicted association has been confirmed by Ref. [1].

e: if the column is blank, it means the prediction has not been confirmed

Supp. Tab. 4 The predict prostate cancer-miRNAs with known prostate cancer miRNA associations and the confirmation source

miRNA	possibility	evidence	miRNA	possibility	evidence	miRNA	possibility	evidence
**miR-18a-5p**	0.9995	$	miR-181d-5p	0.9903	$	miR-186-5p	0.9759	[66]
**miR-155-5p**	0.9994	$	miR-34b-5p	0.9899	#,$	miR-151a-3p	0.9753	#

**miR-20b-5p**	0.9991		miR-181c-5p	0.9899	$	miR-615-3p	0.9743	[67]
**miR-143-3p**	0.9988	$	miR-338-3p	0.9897	[56]	miR-211-5p	0.9741	
**miR-29c-3p**	0.9986	[44]	miR-7-5p	0.9896	$	miR-520e	0.9734	
**miR-195-5p**	0.9986	*,#,$	miR-99a-5p	0.9892	*,#	miR-339-5p	0.9726	
**miR-15b-5p**	0.9986	#,$	let-7d-5p	0.9892	*	miR-196b-5p	0.9718	#
**miR-29a-3p**	0.9984	*,#	miR-215-5p	0.9892	[54]	miR-130a-3p	0.9715	#
**miR-92a-3p**	0.9982	*,#	miR-100-5p	0.9890	*,#,$	miR-149-5p	0.9712	*
**miR-19a-3p**	0.9982	[45]	miR-133b	0.9889	#	miR-663a	0.9704	
**miR-19b-3p**	0.9980	*	miR-224-5p	0.9887	*,#,$	miR-144-3p	0.9703	[68]
**miR-34a-5p**	0.9979	*,#,$	miR-204-5p	0.9884	$	miR-449b-5p	0.9695	[69]
**miR-181a-5p**	0.9979	$	miR-451a	0.9881	[57]	miR-10a-5p	0.9692	*
**miR-92b-3p**	0.9976	[46]	miR-142-5p	0.9876		miR-219a-5p	0.9672	[70]
**miR-1-3p**	0.9973	#	miR-22-3p	0.9876	[58]	miR-10b-5p	0.9672	*
**miR-124-3p**	0.9973	#,$	miR-449a	0.9874	*,#,$	miR-342-3p	0.9645	[71]
**miR-125b-5p**	0.9973	*,#,$	miR-133a-5p	0.9867	#,$	miR-99b-5p	0.9643	#
**miR-200b-3p**	0.9973	$	miR-210-5p	0.9866	[59]	miR-301b-3p	0.9640	#,$
**let-7b-5p**	0.9971	*,#	miR-26a-5p	0.9866	$	miR-488-5p	0.9637	#
**miR-18b-5p**	0.9970		miR-30d-5p	0.9864	#,$	miR-383-5p	0.9610	
**miR-200a-3p**	0.9969	#	miR-26b-5p	0.9863	*,$	miR-301a-3p	0.9607	[72]
**miR-200c-3p**	0.9966	#	miR-302a-3p	0.9863	$	miR-378a-5p	0.9602	#
**miR-141-3p**	0.9959	*,#,$	miR-335-5p	0.9860	#	miR-137	0.9567	[73]
**let-7a-5p**	0.9959	*,#	miR-148a-3p	0.9859	*,#	miR-139-5p	0.9563	[74]
**miR-196a-5p**	0.9956	[47]	miR-199b-5p	0.9857	*,$	miR-372-5p	0.9559	
**miR-103a-3p**	0.9955	*	miR-212-3p	0.9850	[60]	miR-454-3p	0.9548	
**let-7e-5p**	0.9955	[48]	miR-130b-3p	0.9848	#,$	miR-23a-3p	0.9546	*,$
**miR-150-5p**	0.9953	[49]	miR-148b-3p	0.9848		miR-330-3p	0.9524	*,#,$
**miR-107**	0.9948	#	miR-95-5p	0.9843	[61]	miR-324-5p	0.9522	
**miR-203a-3p**	0.9947	$	miR-302b-3p	0.9843		miR-433-5p	0.9501	
**miR-31-5p**	0.9942	*,#,$	miR-320a	0.9840	*	miR-185-5p	0.9501	#
**miR-122-5p**	0.9941	#	miR-302c-3p	0.9835		miR-448	0.9499	
**miR-497-5p**	0.9938	*	miR-198	0.9821	*,#	miR-129-5p	0.9418	[75]
**miR-128-2-5p**	0.9937	[50]	miR-128-3p	0.9814	$	miR-331-3p	0.9417	$
**miR-424-5p**	0.9937	[51]	miR-96-5p	0.9812	*,#,$	miR-520h	0.9410	
**let-7c-5p**	0.9936	*,#,$	miR-296-5p	0.9809	*,#	miR-452-5p	0.9363	#
**miR-126-3p**	0.9929	[52]	miR-206	0.9808		miR-134-5p	0.9317	
**miR-27a-3p**	0.9927	*,#	miR-302d-3p	0.9805		miR-491-5p	0.9285	
**miR-218-5p**	0.9927	*,#,$	miR-484	0.9798	[62]	miR-33b-5p	0.9227	
**miR-30a-5p**	0.9926	*,$	miR-184	0.9791	*	miR-421	0.9212	$
**miR-193b-3p**	0.9925	[53]	miR-197-3p	0.9786	[63]	miR-216a-5p	0.9209	[76]
**miR-9-5p**	0.9922	[54]	miR-520a-3p	0.9777		miR-216b-5p	0.9157	
**miR-98-5p**	0.9921	#	miR-340-5p	0.9776	[64]	miR-217	0.9128	
**miR-30b-5p**	0.9914	*,$	miR-135a-5p	0.9775	*	miR-598-5p	0.9114	[77]
**miR-429**	0.9914	[55]	miR-367-3p	0.9772	[65]	miR-532-5p	0.9034	
**let-7f-5p**	0.9912	*	miR-33a-5p	0.9771		miR-365a-3p	0.9007	

**miR-27b-3p**	0.9910	*,#	miR-153-3p	0.9767	#,$	miR-377-3p	0.8977	[78]
**miR-183-5p**	0.9905	*,#,$	miR-520b	0.9762		miR-499a-5p	0.8926	
**miR-125a-5p**	0.9905	*	miR-140-5p	0.9761		miR-658	0.8923	
**miR-375**	0.9904	*,#	miR-30e-5p	0.9759	$	miR-423-3p	0.8895	[79]

Supp. Tab. 5 The predict breast cancer-miRNAs without known breast cancer miRNA associations and the confirmation source

**miRNA**	**possibility**	**evidence**	**miRNA**	**possibility**	**evidence**	**miRNA**	**possibility**	**evidence 3**
**miR-21-5p**	0.9997	*,#,$	let-7c-5p	0.9826	#,$	miR-22-3p	0.9518	*,#,$
**miR-17-5p**	0.9986	#,$	miR-203a-3p	0.9812	*,#,$	miR-320a	0.9511	*,#
**miR-20a-5p**	0.9979	*,#,$	miR-107	0.9807	#,$	miR-194-5p	0.9510	#
**miR-18a-5p**	0.9978	*,#,$	miR-192-5p	0.9804	[5]	miR-148a-3p	0.9507	*,#,$
**miR-106b-5p**	0.9974	#	miR-34c-5p	0.9799	#,$	miR-204-5p	0.9506	*,#,$
**miR-155-5p**	0.9973	*,#,$	miR-146b-5p	0.9782	*,#,$	miR-95-5p	0.9505	[5]
**miR-146a-5p**	0.9972	$	miR-98-5p	0.9781	*,$	miR-210-5p	0.9498	#
**miR-223-3p**	0.9968	#	miR-497-5p	0.9775	*,#,$	miR-484	0.9493	[16]
**miR-29b-3p**	0.9966	*,#,$	let-7f-5p	0.9772	*,#,$	miR-199b-5p	0.9490	#
**miR-29c-3p**	0.9965	*,#,$	miR-122-5p	0.9770	#,$	miR-142-5p	0.9490	$
**miR-20b-5p**	0.9964	#,$	miR-150-5p	0.9770	$	miR-373-3p	0.9461	*,#,$
**miR-221-3p**	0.9961	*,#,$	miR-30c-5p	0.9766	[80]	miR-212-3p	0.9459	[13]
**miR-92a-3p**	0.9961	#,$	miR-218-5p	0.9763	#,$	miR-148b-3p	0.9458	#
**miR-15a-5p**	0.9961	#,$	miR-424-5p	0.9763	[8]	miR-130b-3p	0.9454	[14]
**miR-143-3p**	0.9960	*,#,$	miR-128-2-5p	0.9759	[7]	miR-367-3p	0.9442	#
**miR-195-5p**	0.9958	*,#,$	miR-27a-3p	0.9752	*,#,$	miR-198	0.9426	
**miR-93-5p**	0.9957	#	miR-127-3p	0.9750	$	miR-128-3p	0.9402	#,$
**miR-15b-5p**	0.9955	[4]	miR-9-5p	0.9746	#	miR-363-3p	0.9367	[15]
**miR-29a-3p**	0.9946	#,$	miR-30a-5p	0.9744	#,$	miR-340-5p	0.9362	#
**miR-222-3p**	0.9945	*,#,$	miR-193b-3p	0.9743	*,#,$	miR-197-3p	0.9337	#,$
**miR-16-5p**	0.9943	#,$	miR-126-3p	0.9736	*,#,$	miR-135b-5p	0.9335	#
**miR-145-5p**	0.9941	*,#,$	miR-375	0.9729	[11]	miR-33a-5p	0.9325	[18]
**miR-24-3p**	0.9939	#,$	miR-215-5p	0.9725	#	miR-96-5p	0.9318	*,#,$
**miR-19a-3p**	0.9938	#,$	let-7d-5p	0.9723	*,#,$	miR-30e-5p	0.9273	[19]
**miR-92b-3p**	0.9938	[5]	miR-205-5p	0.9723	*,#,$	miR-196b-5p	0.9260	[81]
**miR-34a-5p**	0.9938	#,$	miR-30b-5p	0.9708	#	miR-615-3p	0.9234	[20]
**miR-181a-5p**	0.9934	*,#,$	miR-181c-5p	0.9694	[10]	miR-153-3p	0.9217	#,$
**miR-19b-3p**	0.9932	#,$	miR-429	0.9693	*,#,$	miR-296-5p	0.9170	#
**miR-106a-5p**	0.9921	[6]	miR-27b-3p	0.9688	[9]	miR-140-5p	0.9156	#,$
**let-7b-5p**	0.9917	#,$	miR-181d-5p	0.9688	*	miR-186-5p	0.9147	[5]
**miR-181b-5p**	0.9915	*,#,$	let-7g-5p	0.9686	#,$	miR-302a-3p	0.9105	#
**miR-1-3p**	0.9904	#,$	miR-183-5p	0.9684	#,$	miR-151a-3p	0.9097	#

**miR-200a-3p**	0.9903	*,#,$	miR-133b	0.9674	#	miR-135a-5p	0.9096	#,$
**miR-124-3p**	0.9903	#,$	miR-23b-3p	0.9647	#	miR-206	0.9090	*,#,$
**miR-32-5p**	0.9903	[5]	miR-7-5p	0.9645	*,#,$	miR-449b-5p	0.9018	[21]
**miR-200b-3p**	0.9901	*,#,$	miR-34b-5p	0.9609	#,$	miR-144-3p	0.9005	[22]
**miR-199a-5p**	0.9900	#	miR-26b-5p	0.9590	#,$	miR-211-5p	0.8997	$
**miR-25-3p**	0.9895	#	miR-224-5p	0.9585	#,$	miR-130a-3p	0.8989	$
**miR-101-3p**	0.9892	#	miR-125a-5p	0.9585	#,$	miR-302b-3p	0.8962	#
**miR-132-3p**	0.9891	#,$	miR-338-3p	0.9582	#	miR-302c-3p	0.8915	#
**let-7a-5p**	0.9887	*,#,$	miR-133a-5p	0.9580	#,$	miR-663a	0.8894	#
**miR-18b-5p**	0.9885	*,#	miR-26a-5p	0.9576	*,#,$	miR-301b-3p	0.8886	#
**miR-141-3p**	0.9884	*,#,$	miR-449a	0.9566	[12]	miR-302d-3p	0.8837	#
**miR-200c-3p**	0.9882	*,#,$	miR-99a-5p	0.9563	$	miR-149-5p	0.8831	*,#,$
**miR-125b-5p**	0.9878	*,#,$	miR-30d-5p	0.9562	#	miR-301a-3p	0.8776	#
**let-7e-5p**	0.9867	$	miR-191-5p	0.9550	*,#,$	miR-10b-5p	0.8740	*,#,$
**miR-196a-5p**	0.9860	*,#,$	miR-182-5p	0.9549	*,#,$	miR-342-3p	0.8726	#
**miR-214-3p**	0.9844	#	miR-100-5p	0.9545	#,$	miR-520a-3p	0.8639	#
**miR-31-5p**	0.9841	*,#,$	miR-335-5p	0.9529	*,#,$	miR-339-5p	0.8626	#,$
**miR-103a-3p**	0.9835	#	miR-451a	0.9525	#,$	miR-330-3p	0.8423	[24]

Supp. Tab. 6 The predict prostate cancer-miRNAs without known prostate cancer miRNA associations and the confirmation source

miRNA	possibility	evidence	miRNA	possibility	evidence	miRNA	possibility	evidence
**miR-21-5p**	0.9999	*,#,$	miR-192-5p	0.9942	[83]	miR-26b-5p	0.9833	*,$
**miR-17-5p**	0.9995	*,#	miR-31-5p	0.9941	*,#,$	miR-142-5p	0.9831	
**miR-18a-5p**	0.9994	$	miR-34c-5p	0.9940	#,$	miR-191-5p	0.9826	*,#
**miR-155-5p**	0.9993	$	miR-203a-3p	0.9937	$	miR-199b-5p	0.9825	*,$
**miR-20a-5p**	0.9992	*,#,$	miR-107	0.9935	#	miR-210-5p	0.9821	[59]
**miR-146a-5p**	0.9992	*,#,$	miR-146b-5p	0.9933	#	miR-451a	0.9819	[57]
**miR-106b-5p**	0.9990	#	miR-27a-3p	0.9931	*,#	miR-367-3p	0.9817	[65]
**miR-223-3p**	0.9989	*,#,$	miR-98-5p	0.9931	#	miR-212-3p	0.9812	[60]
**miR-29b-3p**	0.9989	$	miR-122-5p	0.9928	#	miR-148a-3p	0.9810	*,#
**miR-221-3p**	0.9988	*,#,$	miR-193b-3p	0.9927	[53]	miR-130b-3p	0.9809	#,$
**miR-15a-5p**	0.9988	*,#,$	miR-497-5p	0.9924	*	miR-22-3p	0.9809	[58]
**miR-143-3p**	0.9988	$	let-7f-5p	0.9923	*	miR-95-5p	0.9806	[61]
**miR-92a-3p**	0.9987	*,#	miR-215-5p	0.9923	[54]	miR-148b-3p	0.9805	
**miR-29c-3p**	0.9987	[44]	miR-218-5p	0.9921	*,#,$	miR-128-3p	0.9804	$
**miR-195-5p**	0.9986	*,#,$	miR-424-5p	0.9920	[51]	miR-198	0.9803	*,#
**miR-15b-5p**	0.9986	#,$	miR-30a-5p	0.9920	*,$	miR-373-3p	0.9802	#
**miR-20b-5p**	0.9986		miR-30c-5p	0.9919	*,#,$	miR-363-3p	0.9796	[84]
**miR-145-5p**	0.9984	*,#,$	miR-205-5p	0.9917	*,#,$	miR-484	0.9792	[62]
**miR-16-5p**	0.9984	*,#,$	miR-150-5p	0.9916	[49]	miR-184	0.9785	*
**miR-222-3p**	0.9983	*,#,$	miR-128-2-5p	0.9914	[50]	miR-96-5p	0.9766	*,#,$

**miR-93-5p**	0.9983	#	miR-27b-3p	0.9910	*,#	miR-33a-5p	0.9755	
**miR-34a-5p**	0.9982	*,#,$	let-7d-5p	0.9906	*	miR-197-3p	0.9755	[63]
**miR-92b-3p**	0.9982	[46]	miR-126-3p	0.9906	[52]	miR-296-5p	0.9727	*,#
**miR-24-3p**	0.9982	[82]	miR-429	0.9905	[55]	miR-135b-5p	0.9724	#
**miR-29a-3p**	0.9982	*,#	miR-183-5p	0.9904	*,#,$	miR-186-5p	0.9721	[66]
**miR-181a-5p**	0.9979	$	miR-9-5p	0.9902	[54]	miR-615-3p	0.9721	[67]
**miR-19a-3p**	0.9978	[45]	miR-30b-5p	0.9899	*,$	miR-206	0.9717	
**miR-19b-3p**	0.9976	*	miR-375	0.9899	*,#	miR-30e-5p	0.9705	$
**let-7b-5p**	0.9974	*,#	let-7g-5p	0.9899	*	miR-340-5p	0.9703	[64]
**miR-106a-5p**	0.9973	*,#	miR-181d-5p	0.9896	$	miR-153-3p	0.9699	#,$
**miR-181b-5p**	0.9972	*,#,$	miR-127-3p	0.9891	*,#	miR-196b-5p	0.9693	#
**miR-124-3p**	0.9972	#,$	miR-181c-5p	0.9891	$	miR-10a-5p	0.9681	*
**miR-32-5p**	0.9970	*,#	miR-23b-3p	0.9885	*,#,$	miR-151a-3p	0.9667	#
**miR-200b-3p**	0.9970	$	miR-34b-5p	0.9875	#,$	miR-140-5p	0.9662	
**miR-1-3p**	0.9968	#	miR-7-5p	0.9873	$	miR-449b-5p	0.9658	[69]
**miR-200a-3p**	0.9968	#	miR-125a-5p	0.9868	*	miR-302a-3p	0.9650	$
**miR-199a-5p**	0.9967	*,#	miR-449a	0.9868	*,#,$	miR-10b-5p	0.9647	*
**miR-125b-5p**	0.9967	*,#,$	miR-338-3p	0.9859	[56]	miR-130a-3p	0.9644	#
**miR-132-3p**	0.9966	#	miR-182-5p	0.9858	*,#,$	miR-135a-5p	0.9640	*
**miR-25-3p**	0.9965	*,#,$	miR-26a-5p	0.9858	$	miR-149-5p	0.9629	*
**miR-101-3p**	0.9964	*,#,$	miR-224-5p	0.9857	*,#,$	miR-211-5p	0.9623	
**miR-18b-5p**	0.9964		miR-100-5p	0.9855	*,#,$	miR-339-5p	0.9617	
**let-7a-5p**	0.9963	*,#	miR-99a-5p	0.9848	*,#	miR-144-3p	0.9610	[68]
**miR-200c-3p**	0.9963	#	miR-133b	0.9846	#	miR-302b-3p	0.9598	
**miR-141-3p**	0.9962	*,#,$	miR-30d-5p	0.9845	#,$	miR-219a-5p	0.9596	[70]
**let-7e-5p**	0.9958	[48]	miR-133a-5p	0.9844	#,$	miR-302c-3p	0.9582	
**miR-196a-5p**	0.9952	[47]	miR-320a	0.9840	*	miR-301b-3p	0.9577	#,$
**miR-103a-3p**	0.9947	*	miR-335-5p	0.9839	#	miR-663a	0.9572	
**let-7c-5p**	0.9945	*,#,$	miR-204-5p	0.9837	$	miR-342-3p	0.9540	[71]
**miR-214-3p**	0.9943	*,#	miR-194-5p	0.9834	#	miR-301a-3p	0.9532	[72]

7. The detail of the co-functional pairs and their confirmed common targets

Supp. Tab. 7 The top 50 candidate cross-cancer associated co-functional miRNA pairs and their validate common targets for the original network and our reconstructed network

**Original network**				**Reconstructed network**			
**miRNA1**	miRNA2	targets	literature	miRNA1	miRNA2	targets	literature
**miR-17-5p**	miR-20a-5p	CDKN1A (p21)	[85]	miR-17-5p	miR-20a-5p	CDKN1A (p21)	[85]
**miR-200b-3p**	miR-200c-3p	AP-2α	[86]	miR-200b-3p	miR-200c-3p	AP-2α	[86]
**miR-29a-3p**	miR-29b-3p	LOXL2	[87]	miR-15a-5p	miR-195-5p	Raf1	[94]
**let-7d-5p**	let-7g-5p			miR-19a-3p	miR-19b-3p	CtIP	[88]
**miR-19a-3p**	miR-19b-3p	CtIP	[88]	miR-15b-5p	miR-195-5p	BCL2	[102]
**miR-200b-3p**	miR-429	AP-2α	[86]	miR-17-5p	miR-106b-5p	E2F	[103]
**let-7f-5p**	let-7g-5p			miR-20a-5p	miR-106b-5p	TIMP-2	[109]

**let-7c-5p**	let-7g-5p		[89]	miR-15a-5p	miR-15b-5p	E2F1	[115]
**let-7c-5p**	let-7f-5p	PGC	[90]	miR-93-5p	miR-106b-5p	CIC	[93]
**let-7a-5p**	let-7c-5p	HMGA2	[89]	miR-29a-3p	miR-29c-3p	LOXL2	[87]
**let-7d-5p**	let-7f-5p			miR-141-3p	miR-200a-3p	p38α	[96]
**let-7a-5p**	let-7f-5p	MYC	[91]	miR-200b-3p	miR-429	AP-2α	[86]
**miR-200c-3p**	miR-429	AP-2α	[86]	miR-29b-3p	miR-29c-3p	LOXL2	[87]
**let-7a-5p**	let-7g-5p			miR-20a-5p	miR-20b-5p	RB1CC1/FIP200	[116]
**let-7c-5p**	let-7d-5p			miR-20a-5p	miR-93-5p	MICA/B	[99]
**miR-17-5p**	miR-93-5p	ABCA1	[92]	miR-17-5p	miR-93-5p	ABCA1	[92]
**let-7e-5p**	let-7g-5p			miR-424-5p	miR-497-5p		
**miR-93-5p**	miR-106b-5p	CIC	[93]	miR-103a-3p	miR-107	CDK5R1	[113]
**miR-15a-5p**	miR-195-5p	Raf1	[94]	miR-29a-3p	miR-29b-3p	LOXL2	[87]
**let-7b-5p**	let-7c-5p	Akt2	[95]	miR-27a-3p	miR-27b-3p	retinoid X receptor α	[117]
**miR-141-3p**	miR-200a-3p	p38alpha	[96]	miR-93-5p	miR-20b-5p	STAT3	[118]
**miR-181a-5p**	miR-181b-5p	RASSF1A	[97]	let-7a-5p	let-7c-5p	HMGA2	[89]
**let-7d-5p**	let-7e-5p			miR-15a-5p	miR-16-5p	Bmi-1	[105]
**let-7a-5p**	let-7b-5p	p53	[98]	miR-106b-5p	miR-20b-5p		
**miR-29b-3p**	miR-29c-3p	LOXL2	[87]	let-7d-5p	let-7g-5p		
**let-7a-5p**	let-7d-5p			miR-17-5p	miR-20b-5p	Ephrin-B2 and EPHB4	[119]
**miR-20a-5p**	miR-93-5p	MICA/B	[99]	miR-200c-3p	miR-429	AP-2α	[86]
**let-7e-5p**	let-7f-5p	MMP11	[100]	miR-195-5p	miR-424-5p		
**let-7c-5p**	let-7e-5p	Cox4i1	[101]	let-7c-5p	let-7e-5p	Cox4i1	[101]
**miR-29a-3p**	miR-29c-3p	LOXL2	[87]	miR-181a-5p	miR-181b-5p	RASSF1A	[97]
**miR-15b-5p**	miR-195-5p	BCL2	[102]	miR-15a-5p	miR-424-5p		
**miR-17-5p**	miR-106b-5p	E2F	[103]	miR-16-5p	miR-195-5p		
**let-7a-5p**	let-7e-5p	IL-13	[104]	miR-195-5p	miR-497-5p	Raf-1 and Ccnd1	[111]
**miR-15a-5p**	miR-16-5p	Bmi-1	[105]	miR-16-5p	miR-15b-5p	BCL2	[120]
**miR-146a-5p**	miR-146b-5p	TRAF6 and IRAK1	[106]	let-7f-5p	let-7g-5p		
**miR-16-5p**	miR-195-5p			let-7a-5p	let-7b-5p	p53	[98]
**let-7b-5p**	let-7e-5p			miR-15b-5p	miR-424-5p		
**let-7f-5p**	miR-98-5p			miR-15b-5p	miR-497-5p	Bcl-2	[121]
**miR-221-3p**	miR-222-3p	ARID1A	[107]	let-7a-5p	let-7e-5p	IL-13	[104]
**let-7b-5p**	let-7f-5p			let-7d-5p	let-7f-5p		
**miR-34b-5p**	miR-34c-5p	α-syn	[108]	let-7c-5p	let-7f-5p	PGC	[90]
**miR-20a-5p**	miR-106b-5p	TIMP-2	[109]	let-7b-5p	let-7c-5p	Akt2	[95]
**let-7b-5p**	let-7g-5p	AKT2	[110]	miR-20a-5p	miR-106a-5p	TIMP-2	[109]
**miR-195-5p**	miR-497-5p	Raf-1 and Ccnd1	[111]	let-7e-5p	let-7f-5p	MMP11	[100]
**let-7a-5p**	miR-98-5p			miR-199a-5p	miR-199b-5p	CLTC	[114]
**miR-34a-5p**	miR-34c-5p	p53	[112]	miR-221-3p	miR-222-3p	ARID1A	[107]
**miR-103a-3p**	miR-107	CDK5R1	[113]	let-7a-5p	let-7f-5p	MYC	[91]
**miR-181b-5p**	miR-181c-5p			miR-15a-5p	miR-497-5p		
**miR-199a-5p**	miR-199b-5p	CLTC	[114]	miR-106a-5p	miR-106b-5p	IL-10	[122]
**miR-98-5p**	let-7g-5p			miR-146a-5p	miR-146b-5p	TRAF6 and IRAK1	[106]

Supp. Tab. 8 The candidate multi-non-cancer-disease associated co-functional miRNA pairs and their validate common targets for the reconstructed network

miRNA1	miRNA2	targets	literature	miRNA1	miRNA2	targets	literature
**hsa-miR-29a-3p**	hsa-miR-29b-3p	LOXL2	[87]	hsa-miR-21-5p	hsa-miR-29b-3p	IL6	[125]
**hsa-miR-17-5p**	hsa-miR-20a-5p	CDKN1A (p21)	[85]	hsa-miR-146a-5p	hsa-miR-155-5p	Histone3	[126]
**hsa-miR-29b-3p**	hsa-miR-29c-3p	LOXL2	[87]	hsa-miR-21-5p	hsa-miR-29a-3p		
**hsa-miR-29a-3p**	hsa-miR-29c-3p	LOXL2	[87]	hsa-miR-17-5p	hsa-miR-21-5p	STAT3	[127]
**hsa-miR-15a-5p**	hsa-miR-15b-5p	E2F1	[115]	hsa-miR-20a-5p	hsa-miR-21-5p	TGF-b	[128]
**hsa-miR-21-5p**	hsa-miR-155-5p	SHIP-1	[123]	hsa-miR-1-3p	hsa-miR-155-5p		
**hsa-miR-21-5p**	hsa-miR-146a-5p	EBNA2	[124]				

8. Supplementary codes

This supplementary file contains five matlab functions such as ComKerMat.m, PreDisRNA.m, DMMD.m, CoFunScore.m and CoFunScoreAll.m. ComKerMat.m and PreDisRNA.m are used to predict disease-miRNA associations. The PreDisRNA.m is the main interface. The other three functions can be used to prioritizing the multi-disease associated co-functional miRNA pairs. The CoFunScoreAll.m is the main interface. One can copy these five functions to five matlab code files.

Function 1: ComKerMat.m

```
%% this function is to compute the kernel matrixes
Function [train_model, test_kernel_matrix] = ComKerMat(ds, ms, train_data, train_label, test_data)
% train_model-- train_model  is the training model of the SVM;
% test_kernel_matrix-- test_kernel_matrix is the output kernel_matrix of the testing dataset;
% ds--the similarity matrix of diseases, the data has not been submitted as the data file is so big
%      (more than 100M is the excel file), one can contact Hui Peng
%     (email:hui.peng-2@student.uts.edu.au);
% ms--the similarity matrix of miRNAs, the data has not been submitted;
% train_data--train_data is the training dataset;
% train_label--train_label is the training labels;
% test_data--test_data is the testing dataset.
train_disease=train_data(:,1);
train_rna=train_data(:,2);
[L1,a]=size(train_data);
test_disease=test_data(:,1);
test_rna=test_data(:,2);
[L2,a]=size(test_data);
K1=zeros(L1,L1);
K2=zeros(L2,L1);
for i=1:L1
    for j=i:L1
        disease1=train_disease(i,1);
        rna1=train_rna(i,1);
        disease2=train_disease(j,1);
        rna2=train_rna(j,1);
        K1(i,j)=sqrt(ms(rna1,rna2)*ds(disease1,disease2));
```

```
        K1(j,i)=K1(i,j);
    end
end
K11=[(1:length(train_disease))',K1];
train_model=svmtrain(train_label,K11,'-t 4 -b 1 -q 1');
for i=1:L2
    for j=1:L1
        K2(i,j)=sqrt(ds(test_disease(i,1),train_disease(j,1))*ms(test_rna(i,1),train_rna(j,1)));
    end
end
test_kernel_matrix=[(1:length(test_disease))',K2];
```

Function 2: PreDisRNA.m

```
%% this function is to predict disease related miRNA
Function [predict_rna,probability] = PreDisRNA(ds, ms, positive_samples, negative_samples,
test_disease, test_RNAs)
% input: % ds--the similarity matrix of diseases, the data has not been submitted as the data
%             file is so big (more than 100M is the excel file), one can contact Hui Peng
%            (email:hui.peng-2@student.uts.edu.au);
%          ms--the similarity matrix of miRNAs, the data has not been submitted;
%        positive_samples--the positive samples for training dataset,four main positive
%                          samples such as positive_miR, positive_hmdd, positive_miRcancer
%                          and positive_pool can be found in Supplementary file 2;
%        negative_samples--the negative samples for training dataset, the negative samples such
%                          as negative_expression can be found in Supplementary file 2;
%        test_disease--the disease that need to predict its related miRNAs, disease ids
%                      are listed in Supplementary file 2
%        test_RNAs--the miRNAs list, the ids can be found in the Supplementary file 2
% output: predict_rna--predicted disease-miRNA, the first column is the id of the diseases and
%                      the second column is the miRNA id, we default output no more than
%                      100 predicted associations of a given disease
%         probability --the probalities of the predicted disease-miRNA association. If
%                    the probability is 1, then this association is a known association in
%                    the training dataset, or the association is a newly predicted one.

%% create the testing dataset the number of the diseases for test
[L1,a]=size(test_disease);
% the number of miRNAs for test
[L2,a]=size(test_RNAs);
% construct the disease-miRNA pairs
k=1;
for i=1:L1
    for j=1:L2
        dis_miR(k,1:2)=[test_disease(i,1),test_RNAs(j,1)];
        k=k+1;
    end
end
```

```
% find the known associations in the training dataset
known_associations_p=intersect(positive_samples,dis_miR,'rows');
known_associations_n=intersect(negative_samples,dis_miR,'rows');
% find the unknown associations pairs to construct the testing dataset
known=[known_associations_p; known_associations_n];
test_dataset=setdiff(dis_miR,known,'rows');
[L3,a]=size(test_dataset);
test_label=zeros(L3,1);
labels=zeros(L3,100);
decs=zeros(L3,1);
%% prediction for 100 times as we will select the same number
%  of negative_samples with positve_samples to construct balanced training set
for i=1:100
    [L4,a]=size(negative_samples);
    [L5,a]=size(positive_samples);
    train_positive=positive_samples;
    train_negative=negative_samples(randperm(L4,L5),:);
    label_p(1:L5,1)=1;
    label_n(1:L5,1)=0;
    training_data=[train_positive;train_negative];
    training_label=[label_p;label_n];
    [train_model,test_kernel_matrix]=ComKerMat(ds,ms,training_data,training_label,test_dataset);
    [predict_label_P, accuracy_P, dec_values_P] = svmpredict(test_label, test_kernel_matrix, train_model,'
-b 1 -q 1');
    labels(:,i)=predict_label_P;
    decs=decs+dec_values_P(:,1);
end
predict_results=[];
predict_rna=[];
decs=decs/100;
m=1;
for i=1:L3
    s=sum(labels(i,:));
    dec=decs(i,1);
    if s>99
        predict_results(m,1:2)=test_dataset(i,:);
        predict_results(m,3)=dec;
        m=m+1;
    end
end

[N,a]=size(predict_results);
[L6,a]=size(known_associations_p);
if L6==0
    known_pair=[];
    known_probability=[];
else
    known_pair=known_associations_p;
    known_probability(1:L6,1)=1;
end
```

```
if N==0
    predict_rnas=[];
    probability=[];
elseif N<=100
    predict_results=sortrows(predict_results,-3);
    predict_rnas=predict_results(:,1:2);
    probability=predict_results(:,3);
else
    predict_results=sortrows(predict_results,-3);
    predict_rnas=predict_results(1:100,1:2);
    probability=predict_results(1:100,3);
end
predict_rna=[known_pair;predict_rnas];
probability=[known_probability;probability];
```

Function 3: DMMD.m

```
%% this function is to find all the disease related miRNAs and the miRNA associated diseases
function [disease_miR,miRNA_dis]=DMMD(dismir)
% dismir is the known disease-miRNA associations
d=unique(dismir(:,1));
m=unique(dismir(:,2));
[L1,a]=size(d);
[L2,a]=size(m);
for i=1:L1
    disease=d(i,1);
    index=find(dismir(:,1)==disease);
    disease_miR{i,1}=disease;
    disease_miR{i,2}=dismir(index,2);
    disease_miR{i,3}=length(index);
end

for i=1:L2
    mirna=m(i,1);
    index=find(dismir(:,2)==mirna);
    miRNA_dis{i,1}=mirna;
    miRNA_dis{i,2}=dismir(index,1);
    miRNA_dis{i,3}=length(index);
end
```

Function 4: CoFunScore.m

```
%% this function is to compute the co_function score
function [score, P, common_dis] = CoFunScore(RNA_target, Dg_map, pair, disease_miR,
miRNA_dis,L1)
```

```
% RNA_target--RNA_target is the miRNA targets where the first column is the miRNA ids the
%                      second column is the miRNA target gene entrez ids. This data set can be found in
%                      the Supplementary file 7;
% Dg_map--Dg_map is the diseases and their related genes, where the first column is the disease Do
%                 id while the second column are their related genes. This data set can be found in
%                 the Supplementary file 6;
% pair--pair is the miRNA pair that composed of two unique miRNAs
% disease_miR, miRNA_dis--disease_miR and miRNA_dis can be computed with the
%                                         function 'DMMD.m'
% L1--L1 is the total diseases in the network
% score--score stores the cfscore of the miRNA pair
% P--P stores the common genes of the miRNA pair and the possibility
% common_dis--common_dis is the common diseases that associated with both of the two miRNAs. In
%             our codes, the common diseases should have  at least one disease gene which is also a
%             target for the miRNA pair
num=0;
rnas=miRNA_dis(:,1);
for i=1:length(rnas)
    RNAs(i,1)=rnas{i,1};
end
rnas=[];
rnas=RNAs;
for i=1:L1
    dis_mir=disease_miR{i,2};
    index=intersect(dis_mir,pair);
    if length(index)==2
        num=num+1;
    end
end

rna1=pair(1,1);
rna2=pair(1,2);
index1=find(rnas==rna1);
index2=find(rnas==rna2);

dis_set1=miRNA_dis{index1,2};
dis_set2=miRNA_dis{index2,2};
L3=length(dis_set1);
L4=length(dis_set2);
d_genes=[];

disease_sets=intersect(dis_set1,dis_set2);

set1=union(dis_set1,dis_set2);
set2=intersect(dis_set1,dis_set2);

f1=(length(set2)/length(set1))*(length(set2)/L1);
gene1=RNA_target{rna1,2};
gene2=RNA_target{rna2,2};
set3=union(gene1,gene2);
set4=intersect(gene2,gene1);
```

```
f2=length(set4)/length(set3);

common_dis=[];
common_gene=[];
for i=1:length(disease_sets)
    dis=disease_sets(i,1);
    dis_gene=Dg_map{dis,3};
    c_g=intersect(dis_gene,set4);
    if length(c_g)>0
        common_dis=[common_dis;dis];
        common_gene=[common_gene;c_g];
    end
end

if length(common_gene)==0
    score=0;
    P=[];
else
    common_gene=unique(common_gene);
    P=zeros(length(common_gene),2);

    for i=1:length(common_gene)
        gene=common_gene(i,1);
        P(i,1)=gene(1,1);
        com_dis_num=length(common_dis);
        if com_dis_num==0
            P(i,2)=0;
        else
            gene_num=0;
            for j=1:com_dis_num
                dis=common_dis(j,1);
                dis_gene=Dg_map{dis,3};
                index=find(dis_gene==gene);
                if length(index)==1
                    gene_num=gene_num+1;
                end
            end
            P(i,2)=gene_num/com_dis_num;
        end
    end

    P=sortrows(P,-2);
    set5=union(common_gene,set4);
    set6=intersect(common_gene,set4);


    f3=length(set6)/length(set5);
    score=f1*f2*f3;
end
```

Function 5: CoFunScoreAll.m

```
%% this function is to compute all the CoFunScore
function [Scores, Genes, Disease] = CoFunScoreAll(dis_mir, RNA_target, Dg_map, t)
% dis_mir--dis_mir is the disease-miRNA associations in the DGR network, where the first column is the
%              disease id and the second column is the miRNA id, in our experiment, the disease-miRNA
%              associations in the DGR network are stored in the Supplementary file 4.
% RNA_target--RNA_target is the miRNA targets where the first column is the miRNA id the
%                     second column is the miRNA target gene entrez id. This data set can be found in the
%                     Supplementary file 7.
% Dg_map--Dg_map is the diseases and their related genes, where the first column is the disease Do
%               id while the second column is disease name. The disease genes are stored in the third
%               column. This data set can be found in the Supplementary file 6.
% t--t is the threshold of the number of diseases that the co-functional pair associated with. We set t=10 in
%    our experiments.
% Scores--Scores stores the output multi-disease associated co-functional miRNA pairs. The first
%             two columns are the miRNA ids while the last column is the cfScores of these pairs.
% Genes--Genes stores the potential common targets of the co-functional miRNA pairs. The first
%            column is the potential genes of the miRNA pair, the second column is the cfScore of
%            the corresponding miRNA pair.
% Disease--Disease stores the miRNA-pairs associated diseases. The first column is the diseases and
%              the second column is the cfScore of the corresponding miRNA pairs.

[disease_miR,miRNA_dis]=DMMD(dis_mir);
[L1,a]=size(disease_miR);
[L2,a]=size(miRNA_dis);
k=1;
rnas=[];
for i=1:L2
    if miRNA_dis{i,3}>=t
        rnas(k,1)=miRNA_dis{i,1};
        k=k+1;
    end
end
if length(rnas)>0
    Pairs_all=combntns(rnas,2);
    [N,a]=size(Pairs_all);
    m=1;
    for i=1:N
        pair=Pairs_all(i,:);
        [score,gene,diseases]=CoFunScore(RNA_target,Dg_map,pair,disease_miR,miRNA_dis,L1);
        if length(diseases)>=t
            Scores(m,1:2)=pair;
            Scores(m,3)=score;
            Genes{m,2}=score;
            Genes{m,1}=gene;
            Disease{m,1}=diseases;
            Disease{m,2}=score;
```

```
            m=m+1;
        end
    end

    if length(Scores)==0
        Scores=[];
        Genes=[];
        Disease=[];
    else
        Scores=sortrows(Scores,-3);
        Genes=sortrows(Genes,-2);
        Disease=sortrows(Disease,-2);
    end
else
    Scores=[];
    Genes=[];
    Disease=[];
end
```

Reference

[1] Chen, X. & Yan, G.-Y. Semi-supervised learning for potential human micrornadisease associations inference. Scientific reports 4 (2014).

[2] Xu, J. et al. Prioritizing candidate disease mirnas by topological features in the mirna target-dysregulated network: Case study of prostate cancer. Molecular cancer therapeutics 10, 1857-1866 (2011).

[3] Jiang, Q., Wang, G., Jin, S., Li, Y. & Wang, Y. Predicting human micrornadisease associations based on support vector machine. International journal of data mining and bioinformatics 8, 282-293 (2013).

[4] Kedmi, M. et al. Egf induces micrornas that target suppressors of cell migration: mir-15b targets mtss1 in breast cancer. cancer 6, 8 (2015).

[5] Yang, Z. et al. dbdemc: a database of differentially expressed mirnas in human cancers. BMC genomics 11, S5 (2010).

[6] Zhao, Q. et al. A direct quantification method for measuring plasma micrornas identified potential biomarkers for detecting metastatic breast cancer. (2016).

[7] Qian, P. et al. Loss of snail regulated mir-128-2 on chromosome 3p22. 3 targets multiple stem cell factors to promote transformation of mammary epithelial cells. Cancer research 72, 6036-6050 (2012).

[8] Zhang, L. et al. A circulating mirna signature as a diagnostic biomarker for noninvasive early detection of breast cancer. Breast cancer research and treatment 154, 423-434 (2015).

[9] Takahashi, R.-u. et al. Loss of microrna-27b contributes to breast cancer stem cell generation by activating enpp1. Nature communications 6 (2015).

[10] Lowery, A. J. et al. Microrna signatures predict oestrogen receptor, progesterone receptor and her2/neu receptor status in breast cancer. Breast Cancer Res 11, R27 (2009).

[11] Zehentmayr, F. et al. Hsa-mir-375 is a predictor of local control in early stage breast cancer. Clinical epigenetics 8, 1 (2016).

[12] Shi, W. et al. Mir-449a promotes breast cancer progression by targeting crip2. Oncotarget (2016).

[13] Hanieh, H. Aryl hydrocarbon receptor-microrna-212/132 axis in human breast cancer suppresses metastasis by targeting sox4. Molecular cancer 14, 1 (2015).

[14] Janssen, E. A. et al. Biologic profiling of lymph node negative breast cancers by means of microrna expression. Modern Pathology 23, 1567-1576 (2010).

[15] Zhang, R., Li, Y., Dong, X., Peng, L. & Nie, X. Mir-363 sensitizes cisplatininduced apoptosis targeting in mcl-1 in breast cancer. Medical Oncology 31, 1-10 (2014).

[16] Zearo, S. et al. Microrna-484 is more highly expressed in serum of early breast cancer patients compared to healthy volunteers. BMC cancer 14, 1 (2014).

[17] Phua, Y. W. et al. Microrna profiling of the pubertal mouse mammary gland identifies mir-184 as a candidate breast tumour suppressor gene. Breast Cancer

Res 17, 83 (2015).

[18] Zhang, C. et al. Mir-33a suppresses breast cancer cell proliferation and metastasis by targeting adam9 and ros1. Protein & cell 6, 881-889 (2015).

[19] Lin, Z. et al. Abnormal mirna-30e expression is associated with breast cancer progression. Clinical laboratory 62, 121-128 (2015).

[20] Bai, Y., Li, J., Li, J., Liu, Y. & Zhang, B. Mir-615 inhibited cell proliferation and cell cycle of human breast cancer cells by suppressing of akt2 expression. International journal of clinical and experimental medicine 8, 3801 (2015).

[21] Lau, L., Cheung, Y., Wong, S., Chan, Y. & Khoo, U. Mir-449b increased tamoxifen sensitivity through akt inactivation. In Annual Meeting of the American Association for Cancer Research, AACR 2011 (The American Association for Cancer Research., 2011).

[22] Yu, L. et al. Microrna-144 affects radiotherapy sensitivity by promoting proliferation, migration and invasion of breast cancer cells. Oncology Reports 34, 1845-1852 (2015).

[23] Cui, W. et al. mirna-520b and mir-520e sensitize breast cancer cells to complement attack via directly targeting 3 utr of cd46. Cancer biology & therapy 10, 232-241 (2010).

[24] Jeyapalan, Z. et al. Expression of cd44 3-untranslated region regulates endogenous microrna functions in tumorigenesis and angiogenesis. Nucleic acids research 39, 3026-3041 (2011).

[25] Mishra, S., Srivastava, A. K., Suman, S., Kumar, V. & Shukla, Y. Circulating mirnas revealed as surrogate molecular signatures for the early detection of breast cancer. Cancer letters 369, 67-75 (2015).

[26] Bamodu, O. A. et al. Aberrant kdm5b expression promotes aggressive breast cancer through malat1 overexpression and downregulation of hsa-mir-448. BMC cancer 16, 1 (2016).

[27] Li, X., Zhang, J., Tang, J., Qin, J. & Yao, Y. The diagnostic and clinical value of mir-433 in circulating tumor cell for breast cancer. Int J Clin Exp Med 9, 1272-1280 (2016).

[28] Zhang, J., Ma, Y., Wang, S., Chen, F. & Gu, Y. C/ebpα inhibits proliferation of breast cancer cells via a novel pathway of mir-134/creb. International journal of

clinical and experimental pathology 8, 14472 (2015).

[29] Hui, Z. et al. mir-491-5p functions as a tumor suppressor by targeting jmjd2b in erα-positive breast cancer. FEBS letters 589, 812-821 (2015).

[30] Lin, Y. et al. Microrna-33b inhibits breast cancer metastasis by targeting hmga2, sall4 and twist1. Scientific reports 5 (2015).

[31] Lerebours, F. et al. mirna expression profiling of inflammatory breast cancer identifies a 5-mirna signature predictive of breast tumor aggressiveness. International Journal of Cancer 133, 1614-1623 (2013).

[32] Mar-Aguilar, F. et al. Serum circulating microrna profiling for identification of potential breast cancer biomarkers. Disease markers 34, 163-169 (2013).

[33] Cava, C. et al. Integration of mrna expression profile, copy number alterations, and microrna expression levels in breast cancer to improve grade definition. PloS one 9, e97681 (2014).

[34] Matamala, N. et al. Microrna deregulation in triple negative breast cancer reveals a role of mir-498 in regulating brca1 expression. Oncotarget 7, 20068-20079 (2016).

[35] Kim, J. et al. Microrna signature for her2-positive breast and gastric cancer. Anticancer research 34, 3807-3810 (2014).

[36] Kolacinska, A. et al. Association of micrornas and pathologic response to preoperative chemotherapy in triple negative breast cancer: preliminary report. Molecular biology reports 41, 2851-2857 (2014).

[37] Krishnan, P. et al. Next generation sequencing profiling identifies mir-574-3p and mir-660-5p as potential novel prognostic markers for breast cancer. BMC genomics 16, 1 (2015).

[38] van Schooneveld, E. et al. Expression profiling of cancerous and normal breast tissues identifies micrornas that are differentially expressed in serum from patients with (metastatic) breast cancer and healthy volunteers. Breast Cancer Res 14, R34 (2012).

[39] Mangolini, A. et al. Diagnostic and prognostic micrornas in the serum of breast cancer patients measured by droplet digital pcr. Biomarker research 3, 1 (2015).

[40] Cuk, K. et al. Plasma microrna panel for minimally invasive detection of breast cancer. PloS one 8, e76729 (2013).

[41] Sim, J. et al. High microrna-370 expression correlates with tumor progression and poor prognosis in breast cancer. Journal of breast cancer 18, 323-328 (2015).

[42] Madhavan, D. et al. Circulating mirnas with prognostic value in metastatic breast cancer and for early detection of metastasis. Carcinogenesis bgw008 (2016).

[43] Chen, L. et al. Role of deregulated micrornas in breast cancer progression using ffpe tissue. PloS one 8, e54213 (2013).

[44] Nishikawa, R. et al. Tumor-suppressive microrna-29s inhibit cancer cell migration and invasion via targeting lamc1 in prostate cancer. International journal of oncology 45, 401-410 (2014).

[45] Mo, W. et al. Identification of novel ar-targeted micrornas mediating androgen signalling through critical pathways to regulate cell viability in prostate cancer. PloS one 8, e56592 (2013).

[46] Lodes, M. J. et al. Detection of cancer with serum mirnas on an oligonucleotide microarray. PLoS One 4, e6229 (2009).

[47] Josson, S., Sung, S.-Y., Lao, K., Chung, L. W. & Johnstone, P. A. Radiation modulation of microrna in prostate cancer cell lines. The Prostate 68, 1599-1606 (2008).

[48] Chen, Z.-H. et al. A panel of five circulating micrornas as potential biomarkers for prostate cancer. The Prostate 72, 1443-1452 (2012).

[49] Dezhong, L. et al. mir-150 is a factor of survival in prostate cancer patients. J BUON 20, 173-9 (2015).

[50] Khan, A. P. et al. Quantitative proteomic profiling of prostate cancer reveals a role for mir-128 in prostate cancer. Molecular & Cellular Proteomics 9, 298-312 (2010).

[51] Banyard, J. et al. Regulation of epithelial plasticity by mir-424 and mir-200 in a new prostate cancer metastasis model. Scientific reports 3 (2013).

[52] Song, L., Xie, X., Yu, S., Peng, F. & Peng, L. Microrna-126 inhibits proliferation and metastasis by targeting pik3r2 in prostate cancer. Molecular medicine reports 13, 1204-1210 (2016).

[53] Kaukoniemi, K. M. et al. Epigenetically altered mir-193b targets cyclin d1 in prostate cancer. Cancer medicine 4, 1417-1425 (2015).

[54] Walter, B. A., Valera, V. A., Pinto, P. A. & Merino, M. J. Comprehensive microrna profiling of prostate cancer. J Cancer 4, 350-7 (2013).

[55] Ouyang, Y. et al. Downregulation of microrna-429 inhibits cell proliferation by targeting p27kip1 in human prostate cancer cells. Molecular medicine reports 11, 1435-1441 (2015).

[56] Bakkar, A. et al. microrna 338-3p exhibits tumor suppressor role and its downregulation is associated with adverse clinical outcome in prostate cancer patients. Molecular biology reports 43, 229-240 (2016).

[57] Wang, J. et al. Next generation sequencing of pancreatic cyst fluid micrornas from low grade-benign and high grade-invasive lesions. Cancer letters 356, 404-409 (2015).

[58] Pasqualini, L. et al. mir-22 and mir-29a are members of the androgen receptor cistrome modulating lamc1 and mcl-1 in prostate cancer. Molecular Endocrinology 29, 1037-1054 (2015).

[59] Taddei, M. L. et al. Senescent stroma promotes prostate cancer progression: The role of mir-210. Molecular oncology 8, 1729-1746 (2014).

[60] Yang, Y. et al. Dysregulation of microrna-212 promotes castration resistance via hnrnph1-mediated regulation of ar and ar-v7: Implications for racial disparity of prostate cancer. Clinical Cancer Research clincanres-1606 (2015).

[61] Huang, X. et al. mirna-95 mediates radioresistance in tumors by targeting the sphingolipid phosphatase sgpp1. Cancer research 73, 6972-6986 (2013).

[62] Haj-Ahmad, Y. Potential urinary mirna biomarker candidates for the accurate detection of prostate cancer among benign prostatic hyperplasia patients (2014).

[63] Zhu, J. et al. Screening key micrornas for castration-resistant prostate cancer based on mirna/mrna functional synergistic network. Oncotarget (2015).

[64] Huang, K., Tang, Y., He, L. & Dai, Y. Microrna-340 inhibits prostate cancer cell proliferation and metastasis by targeting the mdm2-p53 pathway. Oncology reports 35, 887-895 (2016).

[65] Srivastava, A. et al. Microrna profiling in prostate cancer-the diagnostic potential of urinary mir-205 and mir-214. PLoS One 8, e76994 (2013).

[66] Erdmann, K. et al. Elevated expression of prostate cancer-associated genes is linked to down-regulation of micrornas. BMC cancer 14, 1 (2014).

[67] Yun, S. J. et al. Urinary micrornas of prostate cancer: virus-encoded hsv1-mirh18 and hsv2-mir-h9-5p could be valuable diagnostic markers. International neurourol-ogy journal 19, 74 (2015).

[68] Gu, H. et al. Hypoxia-responsive mir-124 and mir-144 reduce hypoxia-induced autophagy and enhance radiosensitivity of prostate cancer cells via suppressing pim1. Cancer medicine (2016).

[69] Mortensen, M. M. et al. High mir-449b expression in prostate cancer is associated with biochemical recurrence after radical prostatectomy. BMC cancer 14, 1 (2014).

[70] Yuan, T. et al. Plasma extracellular rna profiles in healthy and cancer patients. Scientific reports 6, 19413 (2016).

[71] Xie, G. & Jiang, R. [non-coding rnas in castration-resistant prostate cancer]. Zhonghua nan ke xue= National journal of andrology 21, 1014-1019 (2015).

[72] Kurisetty, V. et al. The role of mir-301-3p in the regulation of rho gtpases mediated emt signaling in castration resistant prostate cancer. Cancer Research 73, 5329-5329 (2013).

[73] Mongan, N., Nilsson, E., McWilliam, A. & Downes, M. 432 epigenetic silencing of hsa-mir-137 contributes to prostate cancer recurrence by contributing to overexpression of the lysine specific demethylase 1 (kdm1a). European Journal of Cancer 48, 134-134 (2012).

[74] Sánchez, C. A. et al. Exosomes from bulk and stem cells from human prostate cancer have a differential microrna content that contributes cooperatively over local and pre-metastatic niche. Oncotarget 5 (2015).

[75] Xiao, W., Dai, B., Zhu, Y. & Ye, D. Norcantharidin induces autophagy-related prostate cancer cell death through beclin-1 upregulation by mir-129-5p suppression. Tumor Biology 1-6 (2015).

[76] Miyazaki, T. et al. Microrna library-based functional screening identified androgensensitive mir-216a as a player in bicalutamide resistance in prostate cancer. Journal of clinical medicine 4, 1853-1865 (2015).

[77] Corcoran, C., Rani, S. & O'Driscoll, L. mir-34a is an intracellular and exosomal predictive biomarker for response to docetaxel with clinical relevance to prostate cancer progression. The Prostate 74, 1320-1334 (2014).

[78] Formosa, A. et al. Micrornas, mir-154, mir-299-5p, mir-376a, mir-376c, mir-377, mir-381, mir-487b, mir-485-3p, mir-495 and mir-654-3p, mapped to the 14q32.31 locus, regulate proliferation, apoptosis, migration and invasion in metastatic prostate cancer cells. Oncogene 33, 5173-5182 (2014).

[79] Watahiki, A. et al. Plasma mirnas as biomarkers to identify patients with castration-resistant metastatic prostate cancer. International journal of molecular sciences 14, 7757-7770 (2013).

[80] Yen, M.-C. et al. Isolinderalactone enhances the inhibition of socs3 on stat3 activity by decreasing mir-30c in breast cancer. Oncology reports 35, 1356-1364 (2016).

[81] Marquez, R. T., Smith, A. R., Tsao, B. & Xu, L. Epigenetic dysregulation of mir-196b in breast cancer. Cancer Research 74, 5240-5240 (2014).

[82] Lynch, S. M., McKenna, M. M., Walsh, C. P. & McKenna, D. J. mir-24 regulates cdkn1b/p27 expression in prostate cancer. The Prostate (2016).

[83] Sun, J. et al. mir-192 suppresses the tumorigenicity of prostate cancer cells by targeting and inhibiting nin one binding protein. International journal of molecular medicine 37, 485-492 (2016).

[84] Chen, Y. et al. Microrna 363 mediated positive regulation of c-myc translation affect prostate cancer development and progress. Neoplasma 62, 191-198 (2014).

[85] Mestdagh, P. et al. The mir-17-92 microrna cluster regulates multiple components of the tgf-β pathway in neuroblastoma. Molecular cell 40, 762-773 (2010).

[86] Wu, Y. et al. A mir-200b/200c/429-binding site polymorphism in the 3 untranslated region of the ap-2α gene is associated with cisplatin resistance. PloS one 6, e29043 (2011).

[87] Nishikawa, R. et al. Tumour-suppressive microrna-29s directly regulate loxl2 expression and inhibit cancer cell migration and invasion in renal cell carcinoma. FEBS letters 589, 2136-2145 (2015).

[88] Hühn, D., Kousholt, A., Sørensen, C. S. & Sartori, A. mir-19, a component of the oncogenic mir-17 92 cluster, targets the dna-end resection factor ctip. Oncogene 34, 3977-3984 (2015).

[89] Shell, S. et al. Let-7expression defines two differentiation stages of cancer. Proceedings of the National Academy of Sciences of the United States of America 104, 11400-11405 (2007).

[90] Liu, W.-J. et al. Expression of serum let-7c, let-7i, and let-7f microrna with its target gene, pepsinogen c, in gastric cancer and precancerous disease. Tumor Biology 36, 3337-3343 (2015).

[91] Roush, S. & Slack, F. J. The let-7 family of micrornas. Trends in cell biology 18, 505-516 (2008).

[92] He, Y. et al. Up-regulated mir-93 contributes to coronary atherosclerosis pathogenesis through targeting abca1. International journal of clinical and experimental medicine 8, 674 (2015).

[93] Choi, N. et al. mir-93/mir-106b/mir-375-cic-crabp1: a novel regulatory axis in prostate cancer progression. Oncotarget 6, 23533 (2015).

[94] Wang, F. et al. mir-195 is a key regulator of raf1 in thyroid cancer. OncoTargets and therapy 8, 3021 (2015).

[95] Peng, J., Mo, R., Ma, J. & Fan, J. let-7b and let-7c are determinants of intrinsic chemoresistance in renal cell carcinoma. World journal of surgical oncology 13, 1 (2015).

[96] Mateescu, B. et al. mir-141 and mir-200a act on ovarian tumorigenesis by controlling oxidative stress response. Nature medicine 17, 1627-1635 (2011).

[97] Bräuer-Hartmann,D.etal. Pml/rarα-regulated mir-181a/b cluster targets the tumor suppressor rassf1a in acute promyelocytic leukemia. Cancer research 75, 3411-3424 (2015).

[98] Saleh, A. D. et al. Cellular stress induced alterations in microrna let-7a and let-7b expression are dependent on p53. PLoS One 6, e24429 (2011).

[99] Yang, H. et al. Histone deacetylase inhibitor saha epigenetically regulates mir-17-92 cluster and mcm7 to upregulate mica expression in hepatoma. British journal of cancer 112, 112-121 (2015).

[100] Khella, H. W. et al. Exploring the role of mirnas in renal cell carcinoma progression and metastasis through bioinformatic and experimental analyses. Tumor Biology 33, 131-140 (2012).

[101] Liu, B. et al. Identifying functional mirna-mrna regulatory modules with correspondence latent dirichlet allocation. Bioinformatics 26, 3105-3111 (2010).

[102] Liu, Z. et al. Mir-106b and mir-15b modulate apoptosis and angiogenesis in myocardial infarction. Cellular Physiology and Biochemistry 29, 851-862 (2012).

[103] Trompeter, H.-I. et al. Micrornas mir-17, mir-20a, and mir-106b act in concert to modulate e2f activity on cell cycle arrest during neuronal lineage differentiation of ussc. PloS one 6, e16138 (2011).

[104] Guan, H. et al. Microrna let-7e is associated with the pathogenesis of experimental autoimmune encephalomyelitis. European journal of immunology 43, 104-114 (2013).

[105] Bhattacharya, R. et al. Mir-15a and mir-16 control bmi-1 expression in ovarian cancer. Cancer research 69, 9090-9095 (2009).

[106] Park, H., Huang, X., Lu, C., Cairo, M. S. & Zhou, X. Microrna-146a and microrna-146b regulate human dendritic cell apoptosis and cytokine production by targeting traf6 and irak1 proteins. Journal of Biological Chemistry 290, 2831-2841 (2015).

[107] Yang, Y., Zhao, X. & Li, H. Mir-221 and mir-222 simultaneously target arid1a and enhance proliferation and invasion of cervical cancer cells. European review for medical and pharmacological sciences 20, 1509-1515 (2016).

[108] Kabaria, S., Choi, D. C., Chaudhuri, A. D., Mouradian, M. M. & Junn, E. Inhibition of mir-34b and mir-34c enhances α-synuclein expression in parkinsons disease. FEBS letters 589, 319-325 (2015).

[109] Wang, Z. et al. Oncogenic mir-20a and mir-106a enhance the invasiveness of human glioma stem cells by directly targeting timp-2. Oncogene 34, 1407-1419 (2015).

[110] Kang, W. et al. let-7b/g silencing activates akt signaling to promote gastric carcinogenesis. Journal of translational medicine 12, 281 (2014).

[111] Li, D. et al. Analysis of mir-195 and mir-497 expression, regulation and role in breast cancer. Clinical Cancer Research 17, 1722-1730 (2011).

[112] Kumamoto, K. et al. Nutlin-3a activates p53 to both down-regulate inhibitor of growth 2 and up-regulate mir-34a, mir-34b, and mir-34c expression, and induce senescence. Cancer research 68, 3193-3203 (2008).

[113] Moncini, S. et al. The role of mir-103 and mir-107 in regulation of cdk5r1 expression and in cellular migration. PloS one 6, e20038 (2011).

[114] Aranda, J. F., Canfrán-Duque, A., Goedeke, L., Suárez, Y. & Fernández-Hernando, C. The mir-199-dynamin regulatory axis controls receptor-mediated endocytosis. Journal of cell science 128, 3197-3209 (2015).

[115] Ofir, M., Hacohen, D. & Ginsberg, D. Mir-15 and mir-16 are direct transcriptional targets of e2f1 that limit e2f-induced proliferation by targeting cyclin e. Molecular Cancer Research 9, 440-447 (2011).

[116] Li, S. et al. Mir-20a and mir-20b negatively regulate autophagy by targeting rb1cc1/p200 in breast cancer cells. Life sciences 147, 143-152 (2016).

[117] Ji, J. et al. Over-expressed microrna-27a and 27b influence fat accumulation and cell proliferation during rat hepatic stellate cell activation. FEBS letters 583, 759-766 (2009).

[118] Asadi, S. & Heydari, E. World journal of pharmacy and pharmaceutical sciences (2015).

[119] Wang, W. et al. Preeclampsia up-regulates angiogenesis-associated microrna (ie., mir-17, -20a, and-20b) that target ephrin-b2 and ephb4 in human placenta. The Journal of Clinical Endocrinology & Metabolism 97, E1051-E1059 (2012).

[120] Xia, L. et al. mir-15b and mir-16 modulate multidrug resistance by targeting bcl2 in human gastric cancer cells. International journal of cancer 123, 372-379 (2008).

[121] Nishizawa, T. & Suzuki, H. The role of microrna in gastric malignancy. International journal of molecular sciences 14, 9487-9496 (2013).

[122] Sharma, A. et al. Posttranscriptional regulation of interleukin-10 expression by hsa-mir-106a. Proceedings of the National Academy of Sciences 106, 5761-5766 (2009).

[123] Li, L. et al. Microrna-155 and microrna-21 promote the expansion of functional myeloid-derived suppressor cells. The Journal of Immunology 192, 1034-1043 (2014).

[124] Rosato,P.et al. Dierential regulation of mir-21 and mir-146a by epstein-barr virus-encoded ebna2. Leukemia 26, 2343-2352 (2012).

[125] Patel, S. A. & Gooderham, N. J. Il6 mediates immune and colorectal cancer cell cross-talk via mir-21 and mir-29b. Molecular Cancer Research 13, 1502-1508 (2015).

[126] Doxaki, C., Kampranis, S. C., Eliopoulos, A. G., Spilianakis, C. & Tsatsanis, C. Coordinated regulation of mir-155 and mir-146a genes during induction of endotoxin tolerance in macrophages. The Journal of Immunology 195, 5750-5761 (2015).

[127] Zhang, J. et al. mir-21, mir-17 and mir-19a induced by phosphatase of regenerating liver-3 promote the proliferation and metastasis of colon cancer. British journal of cancer 107, 352-359 (2012).

[128] Valizadeh, M. et al. Down-regulation of tgf-b1, tgf-b receptor 2, and tgf-b associated micrornas, mir-20a and mir-21, in skin lesions of sulfur mustard-exposed Iranian war veterans. Journal of Receptors and Signal Transduction 35, 634-639 (2015).
